# Global burden of burns among children and adolescents: a trend analysis from the global burden of disease study 2019

**DOI:** 10.3389/fpubh.2025.1505023

**Published:** 2025-01-28

**Authors:** Hao Zhu, Ke Wang, Xiong Liu, Jianfeng Ji, Peng Yang, Feng Xu

**Affiliations:** ^1^Department of Emergency Surgery, The First Affiliated Hospital of Soochow University, Suzhou, China; ^2^Department of Intensive Care Unit, Nantong First People's Hospital, Nantong, China; ^3^Burn Unit, Nantong First People's Hospital, Nantong, Jiangsu, China

**Keywords:** children, adolescents, global burden of disease study, burns, trend

## Abstract

**Precise:**

This study investigates global trends in burns among children and adolescents using data from the Global Burden of Disease Study 2019. Analyzing incidence and YLD rates across 204 countries from 1990 to 2019 reveals a general decline in burn-related injuries, with age and sex influencing trends. Notably, in high-SDI regions, incidence rates initially increased before reversing. These findings highlight the need for targeted public health policies and resource allocation to reduce burn-related risks in vulnerable populations.

**Background:**

Given the lack of long-term trend studies on global burns among children and adolescents, this study examined trends in the global burden of burns among children and adolescents.

**Methods:**

The Global Burden of Disease (GBD) Study 2019 provided the statistical data. Sex, age, region, and SDI were used as stratification variables in the study to evaluate the prevalence of burns injuries among kids and teenagers across 204 nations and territories between 1990 and 2019. YLD rates per 100,000 people and incidence rates together with their respective average annual percentage changes (AAPC) were computed to evaluate burden trends. Furthermore, AAPC in YLD rates and incidence rates spanning various age cohorts were analyzed using the Joinpoint software.

**Results:**

The worldwide burns incidence and YLD rates of burns among kids and teenagers exhibited a general declining trend between 1990 and 2019. Concerning the general trend in new cases, incidence rates, and YLD rates, age was positively associated. In 2019, the incidence rate for females surpassed that of males, while YLD rates for females were consistently higher than those of males. New Zealand, Czechia, and Australia rank as the top three among the 204 countries studied. Interestingly, in the high SDI range, the expected values based on incidence rates reversed after first rising with the SDI.

**Conclusion:**

Overall, the incidence and YLD rates of burns among children and adolescents have declined globally, though significant disparities exist across age groups, sexes, regions, and countries. These findings could help guide the development of more targeted strategies to reduce the burn burden in this population.

## Introduction

Burn injuries arise from various sources, including thermal energy, cold, friction, radiation, chemicals, and electricity. Among these, the predominant cause is unintentional exposure to high-temperature agents, such as scalding liquids, heated solids, gases, or radiating heat-emitting objects (1). Burn injuries are an underappreciated trauma that results in lifelong physical and psychological scarring, imposing a substantial burden not only on patients’ families but also on national healthcare systems worldwide (2).

For prevention and allocating resources, information on burn epidemiology is crucial. One of the most thorough and reputable studies offering worldwide epidemiological data on burn injuries is the GBD Study. There is a shortage of comprehensive analysis of the worldwide epidemiology of burns because prior research has frequently been limited to certain nations or areas. Furthermore, inconsistencies and non-standardization in data collection have rendered comparative analysis challenging. Although some studies utilizing Global Burden of Disease (GBD) data on burns have examined global trends in burn burden and conducted multi-level and multi-angle analyses of burn epidemiology, they have primarily concentrated on age-standardized burn incidence rates, Disability-Adjusted Life Years (DALYs), and mortality. These studies predominantly focus on regional differences while neglecting the impact of age factors on burn burden trends (3–8). To the best of our knowledge, no reports of long-term worldwide trends in the epidemiology of burn injuries among minors. Initiatives to lessen the prevalence of noncommunicable illnesses among young people must prioritize treating burn injuries in kids and teenagers. Here, we used the GBD to analyze the trends using 95% uncertainty intervals (UI) and 95% confidence intervals (CI) of the number of incident cases and Years Lived with Disability (YLDs) and their respective rates per 100,000 population, and AAPCs by year and sex for 21 GBD regions, 204 nations and territories from 1990 to 2019.

## Methods

The GBD 2019 provided the data used in this investigation. Estimates for several epidemiological parameters, including years lived with disability (YLDs), years of life lost (YLLs), mortality, incidence, and disability-adjusted life years (DALYs), were provided by the GBD study 2019. These estimates were calculated for 23 age groups and were further stratified by gender (the combined sexes, males, and females). Following their division into 21 regions and seven super-regions, the 204 countries and territories included in the study were examined. Previous publications in other sources have revealed a thorough methodology for the GBD study (9–13). In this study, the burns include three types: 1. Lower airway burns; 2. Burns, <20% total burned surface area without lower airway burns; 3. Burns, > = 20% total burned surface area or > = 10% burned surface area if head/neck or hands/wrist involved w/o lower airway burns.

### Data analysis

Children and adolescents in this study were a diverse group of people ranging in age from 0 to 19. The World Health Organization’s (WHO) definition states that the age of a child is categorized as 0–9 years, while that of an adolescent spans from 10 to 19 years (14). Children are further separated into two age groups: preschool children, ages 0 to 4, and older children, ages 5 to 9. Adolescents are further separated into two age groups: younger adolescents, ages 10 to 14, and older adolescents, ages 15 to 19. The epidemiological outcomes of burns in children and adolescents were analyzed using the Disease Modeling-Meta Regression (DisMod-MR) version 2.1, an extensively used Bayesian meta-regression approach in epidemiological modeling of GBD. The sociodemographic index (SDI) is assigned based on an analysis of a nation’s average years of education, per capita income distribution, and the fertility rate of women under 25. Based on these factors, the 204 countries and territories around the globe are categorized into five SDI levels, ranging from 0 to 1 (low to high).

The number of incident cases, YLDs, and their respective rates, along with AAPCs by year and sex from 1990 to 2019 were all displayed as study results. We used linear regression with year as the independent variable and logarithm-transformed rates as the dependent variable to determine the age- and sex-specific rates as well as their AAPCs. The AAPCs were calculated for the period between 1990 and 2019. The specific calculation methodology for AAPC has been documented in previous studies. The 2.5th and 97.5th values of the ordered 1,000 drawings, which were determined by the GBD method, define the 95% UI. Our study represented the time tendency of incidence and YLD rates by calculating the value of AAPCs and its 95% confidence intervals.

The temporal trends of cases and YLDs due to burns for four age groups and five SDI regions were analyzed by using the Joinpoint regression software. Segmented regression is used in this model to reflect the temporal patterns of disease distribution. For each joinpoint, the APC was calculated, along with a 95% CI. To provide a summary assessment of the general trend across the study, the AAPC was also calculated as a weighted average of the individual APCs. In summary, an increasing trend in the incidence of burns among children and adolescents was indicated if both the APC and the lower 95% CI were higher than 0. In contrast, a declining trend in the incidence of burns was indicated if both values were less than 0. Joinpoint Regression Program version 4.9.0.0 (National Cancer Institute, Rockville, MD, USA) and R version 4.2.2 (R Foundation for Statistical Computing, Vienna, Austria) were used for all statistical analyses and data visualization, and GraphPad Prism 9.0 (GraphPad, Inc., CA, USA). *p* < 0.05 is considered to be statistically significant, and a threshold of *α* = 0.05 was applied.

## Results

### Global burden of burns among children and adolescents

Worldwide, the total amount of incident cases and YLDs dropped from 8,012,944 (95%UI, 6608197–9,883,383) and 995,163 (95%UI, 688540–1,409,058) in 1990 to 7,086,624 (95% UI, 5715161–8,946,820) and 738,026 (95% UI, 513836–1,024,893) in 2019, respectively. Meanwhile, with an AAPC of −0.76 (95% CI, −0.96 to −0.55), the incidence rates dropped from 352.44 (95% UI, 290.65–434.71) per 100,000 people in 1990 to 274.75 (95% UI, 221.58–346.87) in 2019. Moreover, the YLD rates declined from 43.77 (95% UI, 30.28–61.98) per 100,000 people in 1990 to 28.61 (95% UI, 19.92–39.74) in 2019, with the AAPC of −1.41 (95% CI, −1.75 to −1.07) (Table 1).

**Table 1:** The global and regional incidence and years lived with disability and their AAPC from 1990 to 2019.

Characteristics	Incidence						YLDs					
	Counts, 1990	Counts, 2019	Incidence rate (per 100,000 population), 1990	Incidence rate (per 100,000 population), 2019	AAPC, 1990–2019	*P* value	Counts, 1990	Counts, 2019	YLD rate (per 100,000 population), 1990	YLD rate (per 100,000 population), 2019	AAPC, 1990–2019	*P* value
Worldwide	8,012,944 (6608197–9,883,383)	7,086,624 (5715161–8,946,820)	352.44 (290.65–434.71)	274.75 (221.58–346.87)	−0.76 (−0.96 − 0.55)	< 0.001	995,163 (688540–1,409,058)	43.77 (30.28–61.98)	738,026 (513836–1,024,893)	28.61 (19.92–39.74)	−1.41 (−1.75 −1.07)	< 0.001
Sex												
Female	3,859,321 (3128757–4,853,894)	3,439,941 (2734388–4,408,452)	348.06 (282.17–437.76)	275.24 (218.78–352.73)	−1 (−1.63 −0.37)	0.002	510,631 (347972–736,901)	371,962 (258652–515,438)	46.05 (31.38–66.46)	29.76 (20.7–41.24)	−1.47 (−1.75 −1.18)	< 0.001
Male	4,153,623 (3460126–5,076,950)	3,646,682 (2983217–4,517,147)	356.61 (297.07–435.88)	274.3 (224.39–339.77)	−0.82 (−1.03 −0.61)	< 0.001	484,532 (341310–665,740)	366,064 (253323–508,218)	41.6 (29.3–57.16)	27.53 (19.05–38.23)	−1.41 (−1.74 −1.07)	< 0.001
Age group												
<5 years	2,043,841 (1613384–2,593,564)	1,657,158 (1279793–2,143,600)	323.33 (255.24–410.3)	250.01 (193.08–323.39)	−1.11 (−1.75 −0.46)	0.001	76,296 (52137–108,125)	50,292 (33180–71,406)	12.07 (8.25–17.11)	7.59 (5.01–10.77)	−1.56 (−2.24 −0.87)	< 0.001
5–9 years	1,992,177 (1555818–2,577,118)	1,756,494 (1340959–2,339,826)	340.45 (265.88–440.41)	268.29 (204.82–357.39)	−1.02 (−1.62 −0.41)	0.001	211,762 (142008–309,560)	135,824 (91271–192,509)	36.19 (24.27–52.9)	20.75 (13.94–29.4)	−1.87 (−2.13 −1.6)	< 0.001
10–14 years	1,995,731 (1513847–2,639,275)	1,839,312 (1353295–2,508,660)	371.86 (282.07–491.77)	286.41 (210.73–390.64)	−0.79 (−1.05 −0.53)	< 0.001	301,902 (210128–431,866)	218,898 (152559–304,182)	56.25 (39.15–80.47)	34.09 (23.76–47.37)	−1.72 (−1.93 −1.5)	< 0.001
15–19 years	1,981,195 (1503254–2,572,353)	1,833,660 (1352720–2,449,024)	381.29 (289.31–495.06)	295.97 (218.34–395.3)	−0.81 (−1.1 −0.52)	< 0.001	405,202 (281711–561,724)	333,012 (231922–468,821)	77.98 (54.22–108.11)	53.75 (37.43–75.67)	−1.25 (−1.47 −1.03)	< 0.001
SDI region												
High SDI	1,172,381 (943129–1,456,822)	928,046 (735472–1,197,542)	500.67 (402.76–622.14)	420.26 (333.05–542.3)	−0.63 (−0.79 −0.47)	< 0.001	80,054 (47766–127,911)	58,105 (33672–95,107)	34.19 (20.4–54.62)	26.31 (15.25–43.07)	−0.9 (−1.02 −0.78)	< 0.001
High-middle SDI	1,720,274 (1388770–2,162,473)	1,153,778 (916226–1,469,637)	424.5 (342.69–533.61)	351.8 (279.37–448.11)	−0.6 (−1.03 −0.18)	0.005	158,252 (106468–225,416)	77,586 (47686–122,571)	39.05 (26.27–55.62)	23.66 (14.54–37.37)	−1.66 (−1.77 −1.55)	< 0.001
Middle SDI	2,237,058 (1852429–2,750,861)	1,865,148 (1486404–2,368,017)	291.69 (241.54–358.69)	253.23 (201.81–321.5)	−0.14 (−0.8–0.53)	0.69	295,730 (207236–405,359)	176,137 (118458–252,184)	38.56 (27.02–52.86)	23.91 (16.08–34.24)	−1.61 (−1.96 −1.26)	< 0.001
Low-middle SDI	1,611,648 (1322287–1,998,737)	1,596,305 (1288092–1,988,975)	282.45 (231.74–350.29)	229.57 (185.24–286.04)	−1.03 (−1.47 −0.59)	< 0.001	274,320 (198742–365,109)	210,117 (152536–283,419)	48.08 (34.83–63.99)	30.22 (21.94–40.76)	−1.71 (−2.1 −1.33)	< 0.001
Low SDI	1,090,597 (783998–1,665,405)	1,418,557 (1146918–1,789,231)	369.23 (265.43–563.84)	237.63 (192.13–299.72)	−0.62 (−1.18 −0.06)	0.032	185,905 (114041–341,779)	214,997 (149723–299,966)	62.94 (38.61–115.71)	36.02 (25.08–50.25)	−1.85 (−2.3 −1.41)	< 0.001
South–East Asia, East Asia, and Oceania												
East Asia	671,758 (509967–879,172)	463,590 (337066–627,723)	144.32 (109.56–188.88)	149.12 (108.42–201.91)	0.25 (−0.11 −0.61)	0.17	62,703 (41481–91,229)	26,509 (15012–43,364)	13.47 (8.91–19.6)	8.53 (4.83–13.95)	−1.45 (−1.9 −1)	< 0.001
Southeast Asia	626,603 (509283–782,480)	531,248 (420151–682,897)	283.16 (230.15–353.61)	235.65 (186.37 −302.91)	−0.49 (−1.07 −0.1)	0.098	91,471 (63027–130,111)	61,905 (42786–86,249)	41.34 (28.48–58.8)	27.46 (18.98–38.26)	−1.48 (−1.87 −1.08)	< 0.001
Oceania	8,151 (6655–10,136)	15,487 (12525–19,305)	246.23 (201.03–306.2)	253.54 (205.04 −316.04)	−0.14 (−0.66 −0.39)	0.596	1,587 (1120–2085)	2,698 (1937–3,586)	47.94 (33.83–62.99)	44.18 (31.72–58.7)	−0.49 (−0.97–0)	0.049
Sub-Saharan Africa												
Eastern Sub-Saharan Africa	718,603 (459465–1,263,549)	669,418 (518794–871,135)	649.54 (415.31–1142.12)	299.49 (232.1 −389.73)	−2.57 (−5.52 −0.46)	0.095	79,465 (50324–131,910)	80,952 (57868–108,814)	71.83 (45.49–119.23)	36.22 (25.89–48.68)	−2.28 (−2.53 −2.03)	< 0.001
Central Sub-Saharan Africa	77,958 (61444 −104,506)	142,645 (115044–180,640)	247.35 (194.95–331.58)	200.45 (161.67–253.85)	−0.62 (−1.39–0.15)	0.112	20,338 (13176–32,498)	29,099 (20498–40,383)	64.53 (41.81–103.11)	40.89 (28.81–56.75)	−1.46 (−2.33 −0.58)	0.001
Southern Sub-Saharan Africa	57,029 (44358 −73,220)	57,474 (44327–74,803)	217.6 (169.25–279.38)	187.74 (144.79–244.35)	−0.5 (−0.71 −0.29)	< 0.001	5,327 (3542–7,636)	4,651 (3020–6,733)	20.32 (13.52–29.14)	15.19 (9.87–21.99)	−0.97 (−1.1 −0.85)	< 0.001
Western Sub-Saharan Africa	189,183 (153070 −237,592)	387,564 (310026–493,854)	176.25 (142.6–221.34)	156.1 (124.87–198.92)	−0.18 (−0.59–0.23)	0.388	17,887 (12332–24,858)	37,995 (25374–53,828)	16.66 (11.49–23.16)	15.3 (10.22–21.68)	−0.25 (−0.53–0.03)	0.082
South Asia												
South Asia	1,351,407 (1086567–1,691,359)	1,454,215 (1154347–1,827,109)	246.58 (198.26–308.61)	209.38 (166.21–263.07)	−0.77 (−1.19 −0.35)	0.001	226,431 (166195–295,861)	204,077 (147738–271,526)	41.32 (30.32–53.98)	29.38 (21.27–39.1)	−1.14 (−1.73 −0.56)	< 0.001
Latin America and Caribbean												
Caribbean	92,753 (74884–115,061)	93,740 (75091–117,813)	614.74 (496.3–762.58)	603.53 (483.46–758.51)	0.18 (−0.88–1.26)	0.726	15,159 (10920–20,052)	20,518 (13766–30,159)	100.47 (72.37–132.9)	132.1 (88.63–194.17)	1.13 (0.37–1.9)	0.003
Central Latin America	630,288 (502829–818,041)	523,960 (401561–694,113)	765.91 (611.03–994.07)	599.16 (459.2–793.74)	−0.71 (−1.67–0.26)	0.149	93,018 (65347–129,661)	47,424 (30305–71,035)	113.03 (79.41–157.56)	54.23 (34.66–81.23)	−2.4 (−2.88 −1.92)	< 0.001
Tropical Latin America	319,891 (245267–418,458)	191,188 (149623–244,350)	459.39 (352.23–600.94)	286.15 (223.94–365.72)	−1.56 (−1.74 −1.37)	< 0.001	24,669 (15552–36,947)	12,892 (7639–20,177)	35.43 (22.33–53.06)	19.29 (11.43–30.2)	−2.04 (−2.18 −1.9)	< 0.001
Andean Latin America	108,905 (88753–134,817)	107,774 (86090–137,251)	569.12 (463.81–704.53)	455.12 (363.55–579.59)	−0.81 (−0.99 −0.63)	< 0.001	19,681 (14161–25,835)	10,166 (6845–14,656)	102.85 (74–135.01)	42.93 (28.91–61.89)	−2.97 (−3.07 −2.88)	< 0.001
North Africa and Middle East												
North Africa and Middle East	767,922 (623538–949,015)	732,214 (555535–986,780)	425.93 (345.84–526.37)	320.03 (242.81–431.29)	−0.47 (−2.22–1.3)	0.598	143,005 (76216–296,404)	86,767 (51081–145,222)	79.32 (42.27–164.4)	37.92 (22.33–63.47)	−2.57 (−3.05 −2.09)	< 0.001
Central Europe, Eastern Europe, and Central Asia												
Central Asia	207,010 (169814–257,048)	191,129 (152984–242,112)	656.3 (538.38–814.94)	562.43 (450.19–712.46)	−0.99 (−1.22 −0.77)	< 0.001	24,420 (17709–33,160)	15,471 (10416–22,139)	77.42 (56.14–105.13)	45.53 (30.65–65.15)	−1.76 (−1.98 −1.54)	< 0.001
Eastern Europe	511,584 (405914–641,807)	269,635 (211061–349,041)	759.98 (603–953.43)	568.56 (445.05–736)	−0.94 (−1.15 −0.72)	< 0.001	41,552 (27449–60,654)	15,490 (9260–25,060)	61.73 (40.78–90.1)	32.66 (19.53–52.84)	−2.1 (−2.39 −1.8)	< 0.001
Central Europe	341,999 (271038–439,956)	188,461 (147550–242,617)	886.92 (702.9–1140.96)	805.54 (630.68–1037.02)	−0.39 (−0.61 −0.16)	0.001	31,079 (20660–45,637)	11,752 (6908–19,145)	80.6 (53.58–118.35)	50.23 (29.53–81.83)	−1.61 (−1.78 −1.44)	< 0.001
High–income regions												
Southern Latin America	134,772 (109276–167,505)	136,002 (108410–170,881)	695.66 (564.06–864.62)	681.06 (542.89–855.73)	−0.05 (−0.18–0.07)	0.392	15,929 (11060–21,943)	10,328 (6447–15,671)	82.22 (57.09–113.27)	51.72 (32.28–78.47)	−1.57 (−1.63 −1.51)	< 0.001
Western Europe	539,479 (423494–696,811)	439,260 (339229–581,107)	548.22 (430.36–708.11)	476.15 (367.72–629.91)	−0.46 (−0.55 −0.38)	< 0.001	38,406 (22438–62,390)	30,099 (17623–48,812)	39.03 (22.8–63.4)	32.63 (19.1–52.91)	−0.6 (−0.65 −0.55)	< 0.001
North America	316,784 (250533–397,324)	388.92 (307.58–487.8)	255,690 (194857–330,530)	284.29 (216.65–367.5)	−1.22 (−1.88 −0.56)	< 0.001	17,358 (9572–28,761)	14,398 (8012–24,074)	21.31 (11.75–35.31)	16.01 (8.91–26.77)	−1.09 (−1.59 −0.6)	< 0.001
Australasia	61,887 (47523–80,232)	67,274 (51395–88,536)	986.26 (757.35–1278.62)	933.6 (713.23–1228.65)	−0.17 (−0.25 −0.1)	< 0.001	3,841 (2325–6,186)	4,000 (2378–6,463)	61.21 (37.06–98.58)	55.51 (33.01–89.69)	−0.33 (−0.37 −0.29)	< 0.001
Asia Pacific	278,978 (220933–356,177)	553.7 (438.5–706.92)	168,655 (132541–215,460)	522.2 (410.38–667.13)	−0.19 (−0.34 −0.04)	0.014	21,838 (13286–34,316)	10,835 (6138–17,873)	43.34 (26.37–68.11)	33.55 (19.01–55.34)	−0.87 (−0.98 −0.75)	< 0.001

AAPC, average annual percent change; SDI, sociodemographic index; YLDs, years lived with disability. The 95% Uncertainty Interval (UI) is shown in parentheses.

### Trends in the burden of burns among children and adolescents by age

The incidence and YLDs due to burns across all age groups (including kids and teenagers) in 1990 and 2019 are shown in Table 1. The number of worldwide incident cases, YLDs, and their respective rates decreased across the four age groups from 1990 to 2019. From 1990 to 2019, the 15–19 age group exhibited the greatest incidence and YLD rates, with an AAPC of −0.81 (95% CI, −1.1 to −0.52) and −1.25 (95% CI, −1.47 to −1.03), respectively. In contrast, during the same period, the <5 years age group exhibited the lowest incidence and YLD rates. It is observed that with increasing age, the incidence rates, the number of YLDs, and YLD rates increased in 1990 and 2019, despite some fluctuations in the number of incident reports and incidence rates between these years.

**Figure 1:**
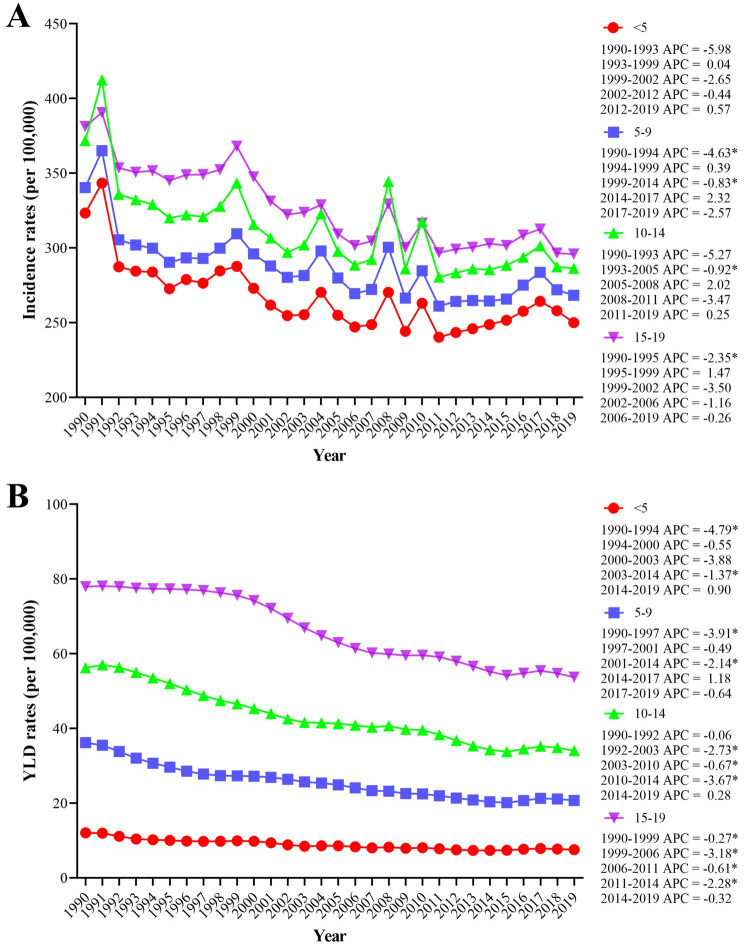
The worldwide incidence rates **(A)** and YLD rates **(B)** of burns resulting from all causes for four age groups between 1990 and 2019 were analyzed using the joinpoint regression algorithm. YLDs = years lived with disability. *p < 0.05.

The incidence rates across the four age groups displayed by the joinpoint regression analysis generally exhibit a downward trend from 1990 to 2019, notwithstanding some fluctuations in certain years. Notably, in some of these years, the age group of 10–14 years old exceeded the age group of 15–19 (Figure 1A). The peak incidence rates across the four age groups all occurred in 1991. In the subsequent years, these rates rebounded and then declined almost simultaneously each time, with no discernible pattern in the intervals between these rebounds. Among the four age groups, the incidence rates in the age group under five exhibited the most significant decrease, with an AAPC of −1.11 (95% CI, −1.75 to −0.46). Compared to the incidence rates, the trend in YLD rates across the four age groups was relatively stable (Figure 1B), particularly for the age group under five. Additionally, for the other three age groups, a clear decreasing trend was observed in certain years. The 5–9 years age group showed the sharpest decrease from 1990 to 1997 (APC: −3.91), the 10–14 years age group from 2010 to 2014 (APC: −3.67), and the 15–19 years age group from 1999 to 2006 (APC: −3.18) (*p* < 0.05).

### Trends in the burden of burns among children and adolescents by sex

**Figure 2:**
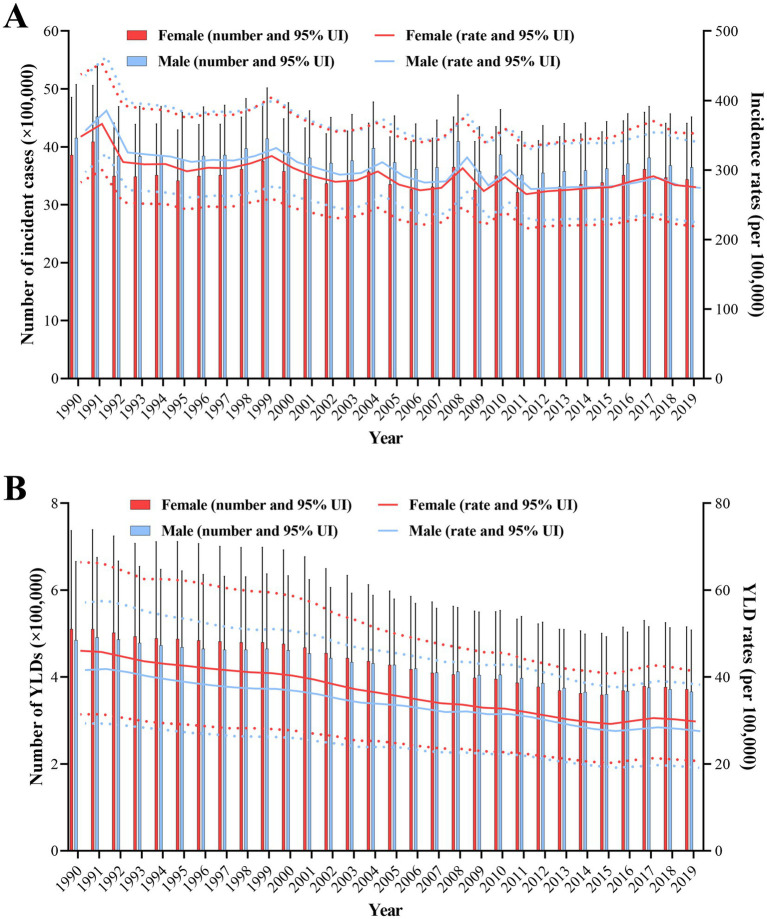
The global number of burns incident cases **(A)**, YLDs **(B)**, and their respective rates resulting from all causes for both males and females from 1990 to 2019 were analyzed by joinpoint regression algorithm. Red and blue dashed line indicates the upper and lower limits of the 95% uncertainty intervals (95% UIs) for females and males, respectively. YLDs, years lived with disability.

Between 1990 and 2019, the number of burns and incidence rates for both men and women have fluctuated but generally exhibited a downward trend (Figure 2A). For males, the number of incident cases decreased from 4,153,623 (95% UI, 3460126–5,076,950) in 1990 to 3,646,682 (95% UI, 2983217–4,517,147) in 2019. Similarly, the incidence rates dropped from 356.61 per 100,000 people (95% UI, 297.07–435.88) in 1990 to 274.3 per 100,000 people(95% UI, 224.39–339.77) in 2019, with an AAPC of −0.82 (95% CI, −1.03 to −0.61). For females, the number of incident cases decreased from 3,859,321 (95% UI, 3128757–4,853,894) in 1990 to 3,439,941 (95% UI, 2734388–4,408,452) in 2019. The incidence rates among females also declined from 348.06 per 100,000 population (95% UI, 282.17–437.76) in 1990 to 275.24 per 100,000 population (95% UI, 218.78–352.73) in 2019, with the AAPC of −1 (95% CI, −1.63 to −0.37) (Table 1). The overall trend in the quantity of YLDs and YLD rates for both males and females exhibited a downward trajectory. The AAPC of males was −1.41 (95% CI, −1.74 to −1.07), while for females it was −1.47 (95% CI, −1.75 to −1.18) (Figure 2B).

### Trends in the burden of burns among children and adolescents by region and nation

**Figure 3:**
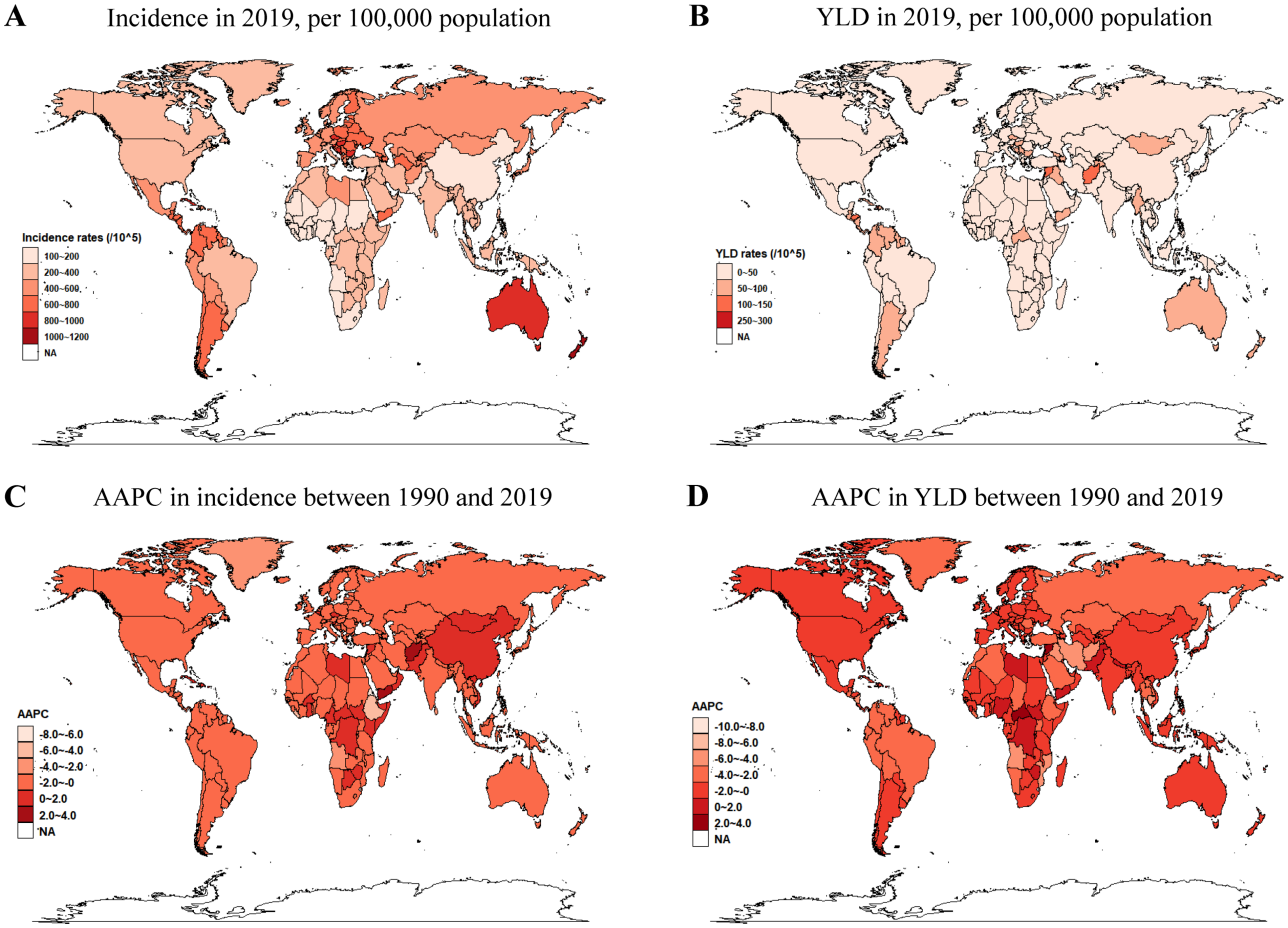
Worldwide diagrams showing incidence rates **(A)**, YLD rates **(B)** in 2019, and AAPC in the incidence rates **(C)**, and YLD rates **(D)** from 1990 to 2019. YLDs, years lived with disability; AAPC, average annual percentage change.

The 204 countries and territories were categorized into 21 GBD regions based on geographical proximity and epidemiological similarities (15). The incidence rates revealed a decreasing trend across all regions, except for the Caribbean, which exhibited an AAPC of 0.18 (95% CI, −0.88 to 1.26), East Asia had an AAPC of 0.25 (95% CI, −0.11 to 0.61), according to Table 1. In 2019, the highest incidence rate was observed in Australasia, reaching 933.6 (95% UI, 713.23 to 1228.65), while East Asia had the lowest incidence rate, at 149.12 (95% UI, 108.42 to 201.91) (Table 1; Figure 3A). The region experiencing the fastest decline in incidence was Eastern Sub-Saharan Africa, with the AAPC of −2.57 (95% CI, −5.52 to 0.46). Conversely, the only two regions that showed an increase in incidence were East Asia, whose AAPC was 0.25 (95% CI, −0.11 to 0.61), the Caribbean, with an AAPC of 0.18 (95% CI, −0.88 to 1.26) (Table 1; Figure 3C). In 2019, the area with the greatest rate of YLD was the Caribbean, significantly surpassing other regions at 132.1 (95% UI, 88.63–194.17). In contrast, East Asia had the lowest YLD rate at 8.53 (95% UI, 4.83–13.95), and this rate continued to decline (Table 1; Figure 3B). The Caribbean is the only region of the GBD regions with a positive increase in the YLD rate, exhibiting an AAPC of 1.13 (95% CI, 0.37–1.9). Conversely, the region with the fastest decline in the YLD rate is Andean Latin America, possessing an AAPC of −2.97 (95% CI, −3.07 to −2.88) (Table 1; Figure 3D).

In 2019, among 204 countries and regions, New Zealand, with an incidence rate of 1033.1 (95% UI, 808.78–1331.3), Czechia, with an incidence rate of 916.27 (95% UI, 718.03–1187.25), and Australia, with an incidence rate of 914.03 (95% UI, 696.71–1200.74) were the three nations with the highest incidence rates. Additionally, the three countries with the fastest increase in incidence rates were Yemen, with an AAPC of 2.97 (95% CI, 1.56–4.4), Afghanistan, with an AAPC of 2.09 (95% CI, −1.72 to 6.05), and Libya, with an AAPC of 1.71 (95% CI, 0.04–3.41). The top three nations in terms of YLD rates were Japan, with a YLD rate of 255.54 (95% UI, 165.74 to 393.34); the Czech Republic, with a YLD rate of 126.39 (95% UI, 60.26–271.84); and Nauru, with a YLD rate of 109.82 (95% UI, 56.06–207.71). The three most rapidly increasing countries in YLD rates were Nauru, with an AAPC of 3.26 (95% CI, 2.10–4.44); Antigua and Barbuda, with an AAPC of 2.96 (95% CI, 2.18–3.74); and Bosnia and Herzegovina, with an AAPC of 1.9 (95% CI, 0.34–3.48). Even though China’s burn incidence rate increased from 144.35 (95% UI, 108.95–189.47) in 1990 to 150.39 (95% UI, 108.95–204.16), with an AAPC of 0.29 (95% CI, −0.08 to 0.67), but the YLD rates decreased from 13.46 (95% UI, 8.93–19.64) to 8.42 (95% UI, 4.67–13.89), with an AAPC of −1.49 (95% CI, −1.96 to −1.02) (Supplementary Table S1).

### Trends in the burden of burns among children and adolescents by SDI

Based on their Sociodemographic Index (SDI) scores, nations can be categorized into five groups. The incidence rates in each region have declined to varying degrees between 1990 and 2019. The fastest drop was from the low-middle SDI region, it’s AAPC is −1.03 (95% CI, −1.47 to −0.59). The greatest incidence rate has consistently been the high SDI region, reaching 500.67 (95% UI, 402.76–622.14) in 1990 and 420.26 (95% UI, 333.05–542.3) in 2019. The highest Years Lived with Disability (YLD) rates have consistently been from the low SDI region, reaching 500.67 (95% UI, 402.76–622.14) in 1990 and 420.26 (95% UI, 333.05–542.3) in 2019. However, the decline rate in the low SDI region was the quickest, with an AAPC of −1.85 (95% CI, −2.3 to −1.41).

**Figure 4:**
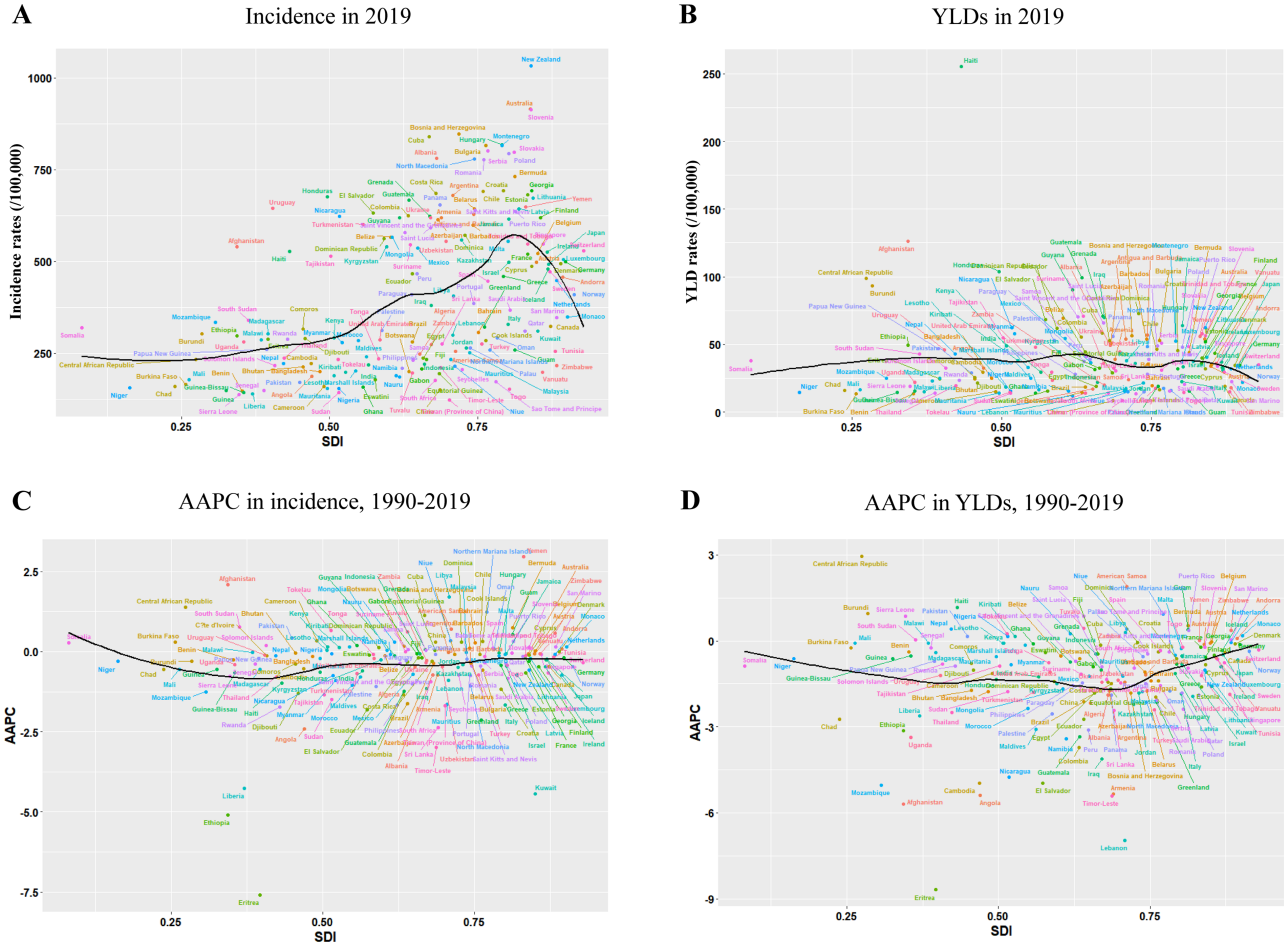
For 204 nations and territories, incidence rates **(A)**, YLD rates **(B)**, AAPC in incidence rates **(C)**, and YLD rates **(D)** by SDI between 1990 and 2019. The predicted values based on incidence rates, YLD rates, AAPC in incidence rates and YLD rates of all SDI regions are indicated by the black solid line. YLDs, years lived with disability; SDI=Sociodemographic Index; AAPC, average annual percentage change.

From the overall trend in 2019, as the SDI index increased, the expected value of the incidence rate also increased; however, in the high SDI range, the expected value dropped sharply (Figure 4A). Except for a small part of the lowest SDI range, which was still increasing, the AAPC expected values of the incidence rate in all other SDI ranges were below 0, indicating a downward trend (Figure 4C). The expected values of YLD rates in all five SDI ranges were relatively stable and did not change significantly (Figure 4B). Although the AAPC predicted values of YLD rates were generally below 0, they decreased as the SDI increased and then rose again in the high-middle SDI range (Figure 4D).

## Discussion

Between 1990 and 2019, even though worldwide incidence and YLD rates of burns in children and adolescents exhibited an overall downward trend, significant differences were observed in the decline rates among the four age groups. This discrepancy highlights the need to investigate the role of age factors in the burden of burns. It is hoped that this study will facilitate the formulation of targeted healthcare policies based on age and region, and provide valuable insights for the development of early intervention measures.

In this study, we found that from 1990 to 2019, the group under five years old exhibited the lowest incidence rate and the fastest rate of drop. This may be because children in this age range receive greater consideration and care from their families and the community, reducing their chances of direct contact with dangerous items. In the same manner, compared to the other groups, this age group’s YLD rate is much lower. As age increases, both the incidence rate and YLD rate also rise, peaking in the 15–19 age group. Previous studies have similarly demonstrated the majority of new cases in 2019 are concentrated in the 10–19 age group (3). This may be attributed to their physiological functions being similar to those of adults, yet lacking sufficient foresight about potential dangers in daily life and work, rendering them more susceptible to burn injuries related to flame, hot liquids, and other hazards. This underscores the need for heightened attention to the 15–19 age group. The causes and risks of burns are diverse, and there are currently no comprehensive global statistics. Existing regional data indicate that hot liquids, cooking and kitchen appliances, heaters and heating equipment, fuels, fuel-burning equipment, heaters, and heating equipment are the most frequent causes of burns in children and adolescents (5, 8, 16–22). Many of these risks can be mitigated through more thorough patient education and frequent reminders.

The role of gender in the incidence and YLD rates is crucial. Our findings show that although the incidence rates for both males and females declined, the burns incidence rate for females has been consistently higher than for males, especially after surpassing male rates in 2016. The YLD rate for women has always been higher than that for males and both have shown a steady decrease for the last 20 years. A previous analysis based on the GBD database for all age groups also highlighted that the DALY rate for females under the age of 19 bears a higher burden than for males in the case of thermal burns (15). The higher incidence rate among children and adolescents for females compared to males may be ascribed to the reality that while the quantity of female incident cases is lower, the overall population of females within this age range is even lower than that of males, leading to a higher incidence rate for female. This is supported by data from the World Population Prospects 2019 Volume II: Demographic Profiles released by the United Nations Population Division. This evidence underscores the need for increased attention to female children and adolescents in health interventions and policy-making.

Among the 21 geographical regions in the GBD study, Australasia has had a consistently high burn incidence. In 2019, New Zealand and Australia ranked first and third, respectively, among 204 countries. While specific causes of burns in children and adolescents in New Zealand are not well documented, data from the Burn Registry of Australia and New Zealand (BRANZ) show that scald injuries accounted for 56.0% of all pediatric burns, with 90.8% occurring at home (23). A study on severe adult burns from the same registry found that most injuries occurred at home (57.5%), with flame burns making up 86% of cases (24). In contrast, China’s average annual percentage change (AAPC) in burn incidence has remained positive over the past 20 years, marking it as one of the few countries with rising burn rates. However, there is no long-term, multicenter study of pediatric burns in China. A study of pediatric burn patients from 2016 to 2019 found hydrothermal scalds to be the most common cause (80.57%), followed by flame burns (3.06%), electricity (1.26%), chemicals (1.25%), other causes (1.48%), and unknown factors (12.39%) (25). Another study from northern China identified the winter months, particularly December, as a peak time for scald injuries, most of which occurred at home (26). To reduce burn incidence in children and adolescents, the focus should be on home safety, especially regarding hot liquids and open flames, with heightened awareness during the cold winter months (27, 28).

The SDI is a composite indicator of development status strongly correlated with health outcomes. It is the geometric mean of 0 to 1 index of total fertility rate under the age of 25 (TFU25), mean education for those ages 15 and older (EDU15+), and lag distributed income (LDI) per capita (29). In 2019, the YLD rates for burns in children and adolescents were not significantly influenced by the SDI, in contrast to adults who are generally more socially active. For adults, YLD rates exhibited a trend inversely related to SDI (3). Although conclusive evidence is lacking, it is hypothesized that this discrepancy may be attributable to the fact that domestic scalds constitute the majority of burn injuries in kids and teenagers globally. Currently, in most regions, irrespective of SDI, disparities in the prevention, treatment, and prognosis of scalds appear to be diminishing (30–36). Finally, it is important to note that China’s SDI in 2019 was 0.686, placing it within the middle SDI zone. Based on global children and adolescent trends in burns incidence and the AAPC in YLD rates by SDI, it is highly likely that the incidence rates of burns and the AAPC in YLD rates among children and adolescents in China may rise in the future. This projection offers an important basis for the development of future policies and the allocation of social resources by relevant authorities.

### Limitations

Despite the utilization of a large global database, several limitations remain. In many regions, children and adolescents with burns may not seek outpatient care at medical institutions, leading to gaps in the gathering of data. Currently, the GBD database lacks comprehensive etiological data on burns in children and adolescents, including clinical details such as complications, severity classifications, and long-term follow-up of prognosis. It is acknowledged that addressing these gaps is a challenging task. Our study utilized only a small fraction of the available data, and the conclusions drawn are consequently somewhat superficial and limited.

## Conclusion

This study examined the global burden of burns among children and adolescents from 1990 to 2019, using data from the Global Burden of Disease Study 2019. Overall, the incidence and YLD rates of burns have shown a declining trend worldwide. However, both incidence and YLD rates were positively associated with age, with higher rates observed in older age groups. Notably, in 2019, the incidence of burns in females surpassed that in males, while YLD rates for females remained consistently higher. Additionally, there were substantial disparities across different regions and countries. The study also highlighted a reversal in expected incidence rates in high SDI regions, where burn incidence initially increased with higher SDI but later declined. These findings underscore the importance of targeted burn prevention strategies and emphasize the need for further research into the region, gender disparities, and age-related trends in burn injuries among children and adolescents.

## Data Availability

The original contributions presented in the study are included in the article/Supplementary material, further inquiries can be directed to the corresponding author/s.
