# Supplementary material for: Global burden of burns among children and adolescents: a trend analysis from the global burden of disease study 2019
Source: Front Public Health. 2025 Jan 28;13:1505023. doi: 10.3389/fpubh.2025.1505023 (PMC11810748; doi:10.3389/fpubh.2025.1505023)
Supplement: Supplementary file 2 [file Supplementary_file_1.doc]

**Table S1. Incidence and years lived with disability of burns and their average annual percentage changes from 1990 to 2019 at the global levels**

AAPC, average annual percent change; YLDs, years lived with disability;The 95% Uncertainty Interval (UI) is shown in parentheses

| **Characteristics** | **Incidence** | | | | | **YLDs** | | | | |
| --- | --- | --- | --- | --- | --- | --- | --- | --- | --- | --- |
|  | **Counts, 1990** | **Counts, 2019** | **incidence rate**  **(per 100 000 Population), 1990** | **incidence rate**  **(per 100 000 Population), 2019** | **AAPC, 1990-2019** | **Counts, 1990** | **Counts, 2019** | **YLD rate**  **(per 100 000 Population), 1990** | **YLD rate**  **(per 100 000 Population), 2019** | **AAPC, 1990-2019** |
| Afghanistan | 26835 (18918-40798) | 116594 (70339-213778) | 419.67 (295.86-638.03) | 539.7 (325.59-989.56) | 2.09 (-1.72 - 6.05) | 44642 (7771-160109) | 27304 (13017-58725) | 698.14 (121.53-2503.9) | 126.39 (60.26-271.84) | -5.69 (-6.07 - -5.3) |
| Albania | 13127 (10292-16959) | 5200 (4065-6685) | 902.19 (707.33-1165.58) | 781.08 (610.5-1004.07) | -0.73 (-1.21 - -0.25) | 1268 (881-1810) | 330 (191-532) | 87.15 (60.55-124.43) | 49.55 (28.71-79.96) | -1.89 (-2.25 - -1.53) |
| Algeria | 38791 (30364-49038) | 36724 (27955-48076) | 287.14 (224.76-362.99) | 248.49 (189.16-325.3) | -0.47 (-1.65 - 0.73) | 4493 (3159-6213) | 2553 (1618-3902) | 33.26 (23.38-45.99) | 17.27 (10.95-26.4) | -2.17 (-2.45 - -1.88) |
| American Samoa | 53 (40-71) | 58 (41-80) | 223.41 (168.51-298.49) | 255.72 (182.18-352.29) | 0.7 (-0.23 - 1.64) | 4 (2-6) | 6 (3-8) | 15.68 (9.82-23.95) | 24.27 (15.12-36.78) | 1.9 (0.34 - 3.48) |
| Andorra | 60 (46-79) | 68 (51-90) | 449.53 (343.13-589.9) | 456.86 (345.63-605.33) | 0.08 (-0.02 - 0.18) | 4 (3-7) | 5 (3-8) | 33.25 (19.08-54.55) | 34.11 (19.82-55.62) | 0.09 (0 - 0.19) |
| Angola | 27702 (17157-50161) | 31891 (25493-40831) | 477.67 (295.85-864.94) | 186.98 (149.47-239.39) | -2.41 (-14.1 - 10.86) | 9853 (4740-20980) | 5557 (3924-7376) | 169.9 (81.73-361.76) | 32.58 (23.01-43.25) | -5.4 (-6.28 - -4.52) |
| Antigua and Barbuda | 168 (136-207) | 149 (118-186) | 687.8 (557.15-847.68) | 627.53 (498.83-785.3) | -0.33 (-0.44 - -0.22) | 20 (14-27) | 14 (9-20) | 80.93 (55.95-111.34) | 57.01 (37.06-86.09) | -1.19 (-1.3 - -1.08) |
| Argentina | 90231 (72862-113078) | 96284 (76763-121002) | 693.17 (559.74-868.68) | 680.34 (542.4-854.99) | -0.02 (-0.13 - 0.09) | 10857 (7546-14988) | 7640 (4857-11422) | 83.41 (57.97-115.14) | 53.99 (34.32-80.7) | -1.45 (-1.6 - -1.3) |
| Armenia | 10144 (8275-12647) | 4796 (3741-6180) | 764.68 (623.8-953.42) | 619.11 (482.92-797.76) | -0.73 (-0.9 - -0.56) | 2553 (1635-3963) | 303 (187-463) | 192.49 (123.24-298.77) | 39.05 (24.11-59.71) | -5.34 (-5.51 - -5.17) |
| Australia | 48461 (37293-62720) | 55040 (41954-72305) | 936.7 (720.83-1212.31) | 914.03 (696.71-1200.74) | -0.07 (-0.12 - -0.02) | 2917 (1745-4730) | 3261 (1941-5236) | 56.38 (33.73-91.42) | 54.16 (32.23-86.95) | -0.13 (-0.15 - -0.11) |
| Austria | 9519 (7481-12269) | 8676 (6634-11473) | 508.03 (399.3-654.81) | 497.67 (380.52-658.09) | -0.07 (-0.15 - 0.01) | 657 (379-1071) | 595 (347-960) | 35.04 (20.24-57.15) | 34.14 (19.88-55.04) | -0.08 (-0.11 - -0.04) |
| Azerbaijan | 21956 (17732-27256) | 18401 (14808-22888) | 700.62 (565.85-869.76) | 613.96 (494.09-763.66) | -0.36 (-1.17 - 0.46) | 2695 (1899-3731) | 1529 (1014-2225) | 85.99 (60.59-119.06) | 51.01 (33.82-74.25) | -1.76 (-1.99 - -1.52) |
| Bahamas | 673 (542-844) | 661 (525-836) | 626.94 (504.96-786.18) | 598.2 (475.36-756.55) | -0.09 (-0.3 - 0.12) | 90 (63-125) | 71 (47-101) | 84.07 (58.73-116.44) | 64.1 (42.8-91.72) | -0.93 (-1.03 - -0.82) |
| Bahrain | 578 (445-748) | 984 (753-1281) | 287.42 (221.33-372.07) | 306.87 (234.69-399.26) | 0.27 (-0.18 - 0.72) | 61 (42-87) | 63 (38-103) | 30.42 (20.73-43.09) | 19.58 (11.79-32.05) | -1.4 (-1.84 - -0.96) |
| Bangladesh | 115543 (92437-145746) | 118816 (93107-152565) | 192.48 (153.99-242.79) | 200.14 (156.83-256.98) | -0.15 (-1.14 - 0.84) | 23401 (16462-31697) | 14767 (10346-20238) | 38.98 (27.42-52.8) | 24.87 (17.43-34.09) | -1.78 (-2.09 - -1.47) |
| Barbados | 508 (405-639) | 415 (325-529) | 595.27 (474.99-748.86) | 598.01 (469.26-763.02) | 0.1 (-0.16 - 0.35) | 64 (43-89) | 37 (24-56) | 74.79 (50.92-103.83) | 53.96 (34.33-80.62) | -1.09 (-1.21 - -0.97) |
| Belarus | 22751 (18330-28558) | 13412 (10563-17078) | 722.3 (581.94-906.66) | 642.58 (506.08-818.25) | -0.37 (-0.44 - -0.3) | 1879 (1254-2783) | 745 (438-1223) | 59.67 (39.8-88.36) | 35.67 (20.98-58.58) | -1.74 (-1.91 - -1.56) |
| Belgium | 13096 (10200-17048) | 13326 (10124-17996) | 529.68 (412.56-689.53) | 520.84 (395.68-703.4) | 0.02 (-0.15 - 0.2) | 891 (517-1461) | 897 (518-1448) | 36.02 (20.92-59.11) | 35.07 (20.24-56.6) | -0.03 (-0.27 - 0.22) |
| Belize | 590 (475-734) | 947 (747-1219) | 581.52 (468.26-722.99) | 566.46 (446.73-729.4) | -0.14 (-0.37 - 0.09) | 82 (58-111) | 116 (79-160) | 80.85 (56.89-109.43) | 69.21 (47.36-95.66) | -0.52 (-0.67 - -0.36) |
| Benin | 4622 (3756-5812) | 10405 (8192-13383) | 160.91 (130.77-202.33) | 146.51 (115.34-188.42) | -0.26 (-0.39 - -0.12) | 440 (306-627) | 964 (619-1411) | 15.33 (10.64-21.83) | 13.57 (8.72-19.86) | -0.38 (-0.55 - -0.22) |
| Bermuda | 95 (71-129) | 87 (64-119) | 598.83 (442.82-812.07) | 730.91 (534.9-999.42) | 0.75 (0.46 - 1.05) | 6 (3-10) | 5 (3-9) | 37.95 (21.05-63.1) | 43.76 (22.78-75.1) | 0.52 (0.35 - 0.68) |
| Bhutan | 655 (520-830) | 564 (444-714) | 200.83 (159.53-254.72) | 213.04 (167.5-269.44) | -0.05 (-0.63 - 0.53) | 131 (92-172) | 64 (45-88) | 40.3 (28.18-52.82) | 24.34 (17.02-33.37) | -1.73 (-2.12 - -1.35) |
| Bolivia (Plurinational State of) | 18170 (14962-22513) | 20648 (16561-26168) | 536.14 (441.47-664.28) | 413.36 (331.54-523.86) | -0.95 (-1.06 - -0.84) | 4035 (2832-5282) | 2499 (1775-3442) | 119.07 (83.57-155.86) | 50.02 (35.54-68.9) | -2.96 (-3.02 - -2.89) |
| Bosnia and Herzegovina | 14016 (11293-17834) | 5626 (4416-7181) | 935.79 (754-1190.7) | 847.61 (665.24-1081.74) | -0.09 (-4.77 - 4.83) | 1521 (1049-2123) | 374 (224-600) | 101.57 (70.06-141.76) | 56.34 (33.73-90.31) | -1.83 (-2.3 - -1.36) |
| Botswana | 1428 (1143-1798) | 1845 (1464-2330) | 197.86 (158.42-249.19) | 200.79 (159.35-253.62) | 0.17 (0.04 - 0.3) | 128 (84-186) | 136 (82-209) | 17.72 (11.61-25.81) | 14.82 (8.97-22.8) | -0.61 (-0.77 - -0.45) |
| Brazil | 310591 (237189-407659) | 180461 (140324-231051) | 459.72 (351.08-603.4) | 281.1 (218.58-359.9) | -1.62 (-1.81 - -1.43) | 23459 (14621-35329) | 11907 (6933-18815) | 34.72 (21.64-52.29) | 18.55 (10.8-29.31) | -2.13 (-2.27 - -1.99) |
| Brunei Darussalam | 652 (514-827) | 658 (518-847) | 566.42 (446.59-718.63) | 501.23 (394.05-644.61) | -0.41 (-0.43 - -0.39) | 64 (43-91) | 52 (32-82) | 55.21 (36.93-79) | 39.89 (24.6-62.49) | -1.11 (-1.18 - -1.03) |
| Bulgaria | 22865 (18022-29080) | 10641 (8328-13646) | 967.93 (762.93-1231.05) | 814.97 (637.83-1045.11) | -0.58 (-0.64 - -0.53) | 1887 (1221-2825) | 693 (409-1116) | 79.89 (51.67-119.58) | 53.1 (31.31-85.47) | -1.39 (-1.47 - -1.31) |
| Burkina Faso | 8531 (6826-10847) | 21199 (16771-27057) | 150.49 (120.41-191.35) | 166 (131.33-211.87) | 0.3 (0.15 - 0.46) | 843 (554-1212) | 1756 (1167-2541) | 14.88 (9.78-21.39) | 13.75 (9.13-19.9) | -0.24 (-0.33 - -0.16) |
| Burundi | 10076 (8142-12632) | 20137 (16047-25692) | 317.59 (256.63-398.17) | 302.34 (240.94-385.76) | -0.31 (-10.37 - 10.87) | 2245 (1608-2967) | 6225 (3904-10541) | 70.76 (50.7-93.51) | 93.46 (58.61-158.27) | 0.95 (0.57 - 1.33) |
| Cabo Verde | 365 (290-465) | 379 (296-487) | 189.82 (150.91-241.67) | 181.28 (141.57-233.17) | -0.1 (-0.24 - 0.04) | 33 (21-48) | 28 (17-43) | 17.07 (10.96-25.07) | 13.21 (7.94-20.79) | -0.87 (-0.95 - -0.79) |
| Cambodia | 15392 (12325-19569) | 14502 (11378-18652) | 266.27 (213.22-338.52) | 222.57 (174.61-286.25) | -0.54 (-1.36 - 0.28) | 7702 (3168-20021) | 1922 (1349-2662) | 133.23 (54.81-346.34) | 29.5 (20.7-40.86) | -4.96 (-5.16 - -4.76) |
| Cameroon | 9055 (7292-11387) | 24357 (19423-31092) | 153.62 (123.71-193.18) | 159.7 (127.35-203.86) | 0.34 (-0.55 - 1.23) | 1247 (807-1768) | 2301 (1509-3313) | 21.16 (13.68-29.99) | 15.08 (9.9-21.72) | -1.13 (-1.25 - -1.02) |
| Canada | 28692 (22749-36163) | 26290 (20429-33737) | 374.26 (296.74-471.7) | 323.33 (251.25-414.92) | -0.47 (-0.56 - -0.38) | 1697 (1025-2766) | 1570 (933-2500) | 22.14 (13.37-36.08) | 19.31 (11.47-30.74) | -0.42 (-0.55 - -0.3) |
| Central African Republic | 2726 (2204-3434) | 6583 (5059-9188) | 181.66 (146.87-228.85) | 233.54 (179.49-325.96) | 1.39 (0.56 - 2.22) | 633 (453-843) | 2785 (1496-5352) | 42.2 (30.18-56.16) | 98.81 (53.08-189.87) | 2.96 (2.18 - 3.74) |
| Chad | 7760 (5660-11466) | 16120 (12999-20246) | 220.33 (160.71-325.55) | 159.76 (128.82-200.65) | -0.56 (-1.01 - -0.1) | 1263 (671-2464) | 1641 (1076-2321) | 35.87 (19.06-69.96) | 16.26 (10.67-23) | -2.74 (-3.18 - -2.29) |
| Chile | 37358 (30654-45795) | 33578 (26710-42440) | 707.71 (580.72-867.54) | 690.33 (549.13-872.53) | -0.09 (-0.42 - 0.24) | 4367 (3009-6056) | 2249 (1320-3587) | 82.74 (57.01-114.72) | 46.24 (27.14-73.75) | -1.95 (-2.16 - -1.74) |
| China | 649295 (490043-852222) | 451051 (326745-612308) | 144.35 (108.95-189.47) | 150.39 (108.95-204.16) | 0.29 (-0.08 - 0.67) | 60564 (40148-88332) | 25243 (14011-41650) | 13.46 (8.93-19.64) | 8.42 (4.67-13.89) | -1.49 (-1.96 - -1.02) |
| Colombia | 139969 (112837-175603) | 93854 (73441-120595) | 926.27 (746.72-1162.09) | 625.25 (489.26-803.39) | -1.35 (-1.65 - -1.04) | 25107 (17916-33335) | 8298 (5142-12506) | 166.15 (118.56-220.6) | 55.28 (34.25-83.32) | -3.72 (-3.82 - -3.61) |
| Comoros | 917 (708-1188) | 963 (717-1281) | 347.23 (268.04-449.83) | 315.99 (235.19-420.34) | -0.38 (-0.72 - -0.04) | 87 (58-122) | 78 (51-118) | 32.87 (21.79-46.24) | 25.75 (16.6-38.62) | -0.79 (-1.02 - -0.55) |
| Congo | 2634 (2122-3362) | 4732 (3750-6038) | 192.34 (154.93-245.51) | 189.41 (150.1-241.64) | -0.28 (-1.09 - 0.55) | 594 (418-789) | 837 (587-1118) | 43.35 (30.52-57.59) | 33.51 (23.48-44.74) | -1.16 (-3.45 - 1.19) |
| Cook Islands | 23 (16-32) | 16 (11-22) | 258.75 (188.84-362.64) | 284.25 (201.93-398.21) | 0.31 (-0.33 - 0.94) | 2 (1-3) | 1 (0-2) | 19.07 (11.36-29.93) | 16.65 (8.89-27.82) | -0.39 (-1.1 - 0.33) |
| Costa Rica | 10647 (8345-13653) | 9710 (7615-12536) | 751.6 (589.05-963.75) | 685.44 (537.52-884.86) | -0.3 (-0.44 - -0.16) | 1151 (769-1655) | 753 (453-1191) | 81.25 (54.31-116.85) | 53.16 (31.98-84.05) | -1.45 (-1.51 - -1.4) |
| Côte te d'Ivoire | 10812.30  (8717.16-13722.81) | 19969.52  (15935.62-25473.95) | 155.72 (125.54-197.63) | 149.86  (119.59-191.17) | -0.13 (-0.38 - 0.12) | 1032 (683-1437) | 1980 (1298-2848) | 14.86 (9.83-20.69) | 14.86 (9.74-21.37) | 0.03 (-0.3 - 0.35) |
| Croatia | 11612 (9055-14937) | 5691 (4497-7262) | 875.62 (682.78-1126.34) | 692.5 (547.25-883.71) | -1.17 (-1.54 - -0.81) | 728 (435-1169) | 372 (222-606) | 54.93 (32.83-88.13) | 45.32 (27.01-73.73) | -0.63 (-0.74 - -0.52) |
| Cuba | 22381 (18105-27680) | 20823 (16207-26905) | 615.37 (497.79-761.07) | 839.96 (653.74-1085.28) | 1.12 (0.91 - 1.34) | 2272 (1514-3287) | 1449 (857-2279) | 62.47 (41.64-90.39) | 58.44 (34.59-91.94) | -0.14 (-0.43 - 0.15) |
| Cyprus | 1309 (1021-1680) | 1357 (1035-1803) | 505.42 (394.05-648.64) | 487.2 (371.5-647.39) | -0.1 (-0.28 - 0.08) | 106 (66-167) | 89 (52-144) | 40.85 (25.3-64.44) | 31.98 (18.69-51.73) | -0.83 (-0.91 - -0.75) |
| Czechia | 27645 (21870-34833) | 19955 (15637-25856) | 908.94 (719.07-1145.28) | 916.27 (718.03-1187.25) | 0.11 (-0.06 - 0.29) | 2138 (1349-3317) | 1228 (714-1997) | 70.28 (44.34-109.07) | 56.37 (32.78-91.71) | -0.71 (-0.83 - -0.59) |
| Democratic People's Republic of Korea | 9989 (8111-12302) | 7315 (5826-9320) | 119.55 (97.07-147.22) | 109.11 (86.9-139.02) | -0.09 (-0.36 - 0.18) | 1144 (772-1568) | 888 (582-1250) | 13.7 (9.24-18.77) | 13.25 (8.69-18.64) | -0.03 (-0.44 - 0.37) |
| Democratic Republic of the Congo | 43289 (35429-53991) | 96416 (77682-124101) | 196.05 (160.45-244.51) | 203.92 (164.3-262.48) | 0.09 (-0.57 - 0.75) | 8923 (6387-11857) | 19462 (13504-27004) | 40.41 (28.93-53.7) | 41.16 (28.56-57.11) | 0.14 (-0.41 - 0.7) |
| Denmark | 6207 (4868-7948) | 6443 (4873-8661) | 496.5 (389.44-635.81) | 494.39 (373.92-664.53) | -0.01 (-0.07 - 0.05) | 445 (259-722) | 450 (260-732) | 35.59 (20.72-57.75) | 34.51 (19.92-56.14) | -0.11 (-0.18 - -0.03) |
| Djibouti | 1072 (826-1401) | 1525 (1136-2044) | 389.93 (300.55-509.92) | 290.57 (216.5-389.52) | -1.3 (-1.81 - -0.78) | 80 (53-118) | 113 (72-169) | 29.24 (19.17-42.92) | 21.58 (13.69-32.18) | -1.01 (-1.31 - -0.7) |
| Dominica | 179 (142-226) | 116 (92-146) | 551.76 (438.36-698.16) | 571.66 (453.46-720.77) | 0.29 (0.12 - 0.45) | 24 (17-33) | 15 (10-21) | 74.19 (52-102) | 72.89 (50.18-101.65) | -0.05 (-0.25 - 0.15) |
| Dominican Republic | 19262 (15528-24199) | 22533 (18052-28238) | 542.39 (437.26-681.42) | 561.32 (449.71-703.44) | 0.13 (0.01 - 0.25) | 3364 (2392-4434) | 2491 (1731-3499) | 94.73 (67.36-124.85) | 62.06 (43.11-87.17) | -1.43 (-1.71 - -1.15) |
| Ecuador | 24576 (19764-30621) | 31207 (24855-39307) | 491.5 (395.28-612.42) | 466.66 (371.68-587.8) | -0.32 (-0.43 - -0.21) | 4996 (3556-6587) | 2974 (1987-4281) | 99.92 (71.11-131.73) | 44.47 (29.71-64.02) | -2.74 (-2.89 - -2.59) |
| Egypt | 70941 (55364-90008) | 99177 (76385-128515) | 253.63 (197.94-321.8) | 236.63 (182.25-306.62) | -0.2 (-0.48 - 0.08) | 9164 (6570-12368) | 7952 (5190-11632) | 32.76 (23.49-44.22) | 18.97 (12.38-27.75) | -1.82 (-2.07 - -1.57) |
| El Salvador | 25840 (19834-35337) | 14474 (11343-18555) | 949.78 (729.02-1298.87) | 631.56 (494.95-809.62) | -1.82 (-3.15 - -0.46) | 8194 (4953-14474) | 1570 (1059-2295) | 301.18 (182.05-532) | 68.5 (46.22-100.16) | -4.97 (-5.27 - -4.67) |
| Equatorial Guinea | 503 (408-635) | 1450 (1150-1871) | 203.08 (164.63-256.54) | 194.23 (153.95-250.59) | 0.04 (-0.22 - 0.29) | 118 (83-153) | 216 (151-295) | 47.76 (33.32-61.59) | 28.99 (20.17-39.46) | -1.68 (-1.82 - -1.55) |
| Eritrea | 34510 (13889-81011) | 9824 (7307-13112) | 1988.37 (800.26-4667.7) | 288.78 (214.79-385.45) | -7.6 (-15.48 - 1.02) | 5790 (2016-14662) | 841 (545-1203) | 333.61 (116.14-844.77) | 24.72 (16.03-35.37) | -8.69 (-9.68 - -7.69) |
| Estonia | 3826 (3132-4715) | 1887 (1481-2454) | 832.6 (681.6-1026.18) | 681.24 (534.86-885.91) | -0.67 (-0.77 - -0.58) | 302 (198-443) | 111 (65-185) | 65.64 (43.19-96.32) | 40.04 (23.43-66.82) | -1.61 (-1.91 - -1.31) |
| Eswatini | 885 (714-1109) | 950 (754-1206) | 185.56 (149.76-232.63) | 178.94 (141.91-227) | -0.11 (-0.29 - 0.07) | 85 (56-121) | 83 (52-121) | 17.83 (11.83-25.47) | 15.61 (9.87-22.83) | -0.41 (-0.53 - -0.29) |
| Ethiopia | 410809 (207124-862264) | 174033 (136894-220084) | 1370.85 (691.16-2877.34) | 296.88 (233.53-375.44) | -5.1 (-9.76 - -0.2) | 36776 (23394-59153) | 28865 (20828-37675) | 122.72 (78.07-197.39) | 49.24 (35.53-64.27) | -3.13 (-3.37 - -2.89) |
| Fiji | 849 (686-1067) | 801 (646-1004) | 236.33 (190.79-296.88) | 234.15 (188.93-293.5) | -0.19 (-0.48 - 0.1) | 137 (97-183) | 111 (77-150) | 38.11 (26.98-50.92) | 32.38 (22.65-44.01) | -0.46 (-0.6 - -0.31) |
| Finland | 8947 (7037-11706) | 7250 (5442-9791) | 704.8 (554.36-922.16) | 619.15 (464.78-836.13) | -0.47 (-0.67 - -0.26) | 633 (370-1025) | 509 (296-815) | 49.84 (29.14-80.73) | 43.46 (25.26-69.61) | -0.5 (-0.71 - -0.3) |
| France | 88641 (70090-115503) | 82742 (63600-109624) | 549.33 (434.36-715.79) | 519.4 (399.24-688.15) | -0.19 (-0.24 - -0.14) | 6260 (3648-10067) | 5775 (3389-9357) | 38.8 (22.61-62.39) | 36.25 (21.27-58.74) | -0.23 (-0.33 - -0.13) |
| Gabon | 1104 (888-1397) | 1574 (1242-2014) | 212.62 (171.08-269.02) | 207.01 (163.44-264.88) | -0.06 (-0.23 - 0.11) | 216 (152-286) | 241 (167-326) | 41.52 (29.35-55.16) | 31.71 (22.02-42.85) | -0.91 (-0.99 - -0.83) |
| Gambia | 922 (736-1173) | 1839 (1434-2399) | 161.25 (128.85-205.25) | 155.31 (121.16-202.61) | -0.2 (-0.4 - 0) | 107 (70-157) | 180 (117-265) | 18.76 (12.17-27.45) | 15.18 (9.86-22.4) | -0.62 (-0.83 - -0.41) |
| Georgia | 13177 (10673-16587) | 6384 (5151-7967) | 734.26 (594.72-924.28) | 692.57 (558.85-864.29) | -0.31 (-0.93 - 0.32) | 1143 (765-1681) | 479 (312-707) | 63.71 (42.63-93.66) | 51.98 (33.81-76.71) | -0.57 (-0.86 - -0.28) |
| Germany | 91550 (71998-118114) | 78517 (60405-105057) | 528.03 (415.26-681.24) | 499.92 (384.6-668.89) | -0.19 (-0.24 - -0.14) | 6279 (3639-10394) | 5444 (3159-8868) | 36.21 (20.99-59.95) | 34.66 (20.11-56.46) | -0.13 (-0.21 - -0.05) |
| Ghana | 12751 (10168-15954) | 23003 (18022-29521) | 153.73 (122.6-192.35) | 157.61 (123.48-202.27) | 0.19 (-0.02 - 0.39) | 1232 (806-1741) | 2089 (1326-3040) | 14.85 (9.72-20.99) | 14.31 (9.09-20.83) | -0.11 (-0.28 - 0.06) |
| Greece | 14702 (11551-19076) | 9059 (6923-12010) | 523.13 (411.01-678.74) | 459.29 (351-608.94) | -0.42 (-0.55 - -0.29) | 1145 (661-1874) | 634 (368-1042) | 40.74 (23.53-66.69) | 32.14 (18.66-52.86) | -0.82 (-0.9 - -0.74) |
| Greenland | 105 (87-125) | 49 (39-60) | 588.56 (488.94-701.49) | 322.16 (257.88-396.56) | -2.14 (-2.26 - -2.03) | 6 (4-10) | 3 (2-5) | 35.84 (21.18-55.9) | 19.06 (10.9-30.58) | -2.16 (-2.3 - -2.01) |
| Grenada | 245 (195-308) | 193 (154-242) | 600.35 (478.77-754.34) | 622.76 (498.84-783.21) | 0.07 (-0.12 - 0.27) | 39 (28-52) | 22 (15-31) | 96.46 (68.8-128.49) | 71.09 (48.31-101.05) | -1.01 (-1.17 - -0.85) |
| Guam | 122 (91-164) | 153 (112-211) | 226.08 (168.94-301.93) | 259.75 (189.93-359.37) | 0.54 (0.26 - 0.81) | 8 (4-12) | 9 (5-15) | 13.99 (8.09-22.61) | 15.56 (8.7-25.44) | 0.39 (0.22 - 0.55) |
| Guatemala | 44093 (35379-55903) | 51533 (40964-65156) | 989.22 (793.71-1254.18) | 666.93 (530.14-843.23) | -1.24 (-1.45 - -1.03) | 11834 (8134-17423) | 7663 (5353-10465) | 265.5 (182.48-390.87) | 99.17 (69.27-135.44) | -3.34 (-3.43 - -3.24) |
| Guinea | 5749 (4677-7215) | 10422 (8269-13266) | 168.76 (137.28-211.8) | 148.07 (117.47-188.47) | -0.56 (-0.83 - -0.3) | 595 (407-842) | 1021 (706-1446) | 17.45 (11.96-24.72) | 14.51 (10.04-20.54) | -0.61 (-0.8 - -0.41) |
| Guinea-Bissau | 963 (791-1211) | 1431 (1130-1815) | 165.28 (135.74-207.83) | 142.89 (112.86-181.25) | -0.66 (-1.01 - -0.31) | 104 (72-146) | 155 (105-220) | 17.85 (12.35-24.98) | 15.46 (10.46-21.98) | -0.52 (-0.68 - -0.36) |
| Guyana | 2392 (1952-2962) | 1744 (1424-2149) | 643.12 (524.73-796.19) | 619 (505.48-762.99) | -0.12 (-0.57 - 0.34) | 419 (302-558) | 254 (179-343) | 112.64 (81.08-150.04) | 90.17 (63.67-121.77) | -0.73 (-0.87 - -0.58) |
| Haiti | 23260 (19082-28703) | 29018 (23800-35530) | 699.55 (573.89-863.24) | 527.47 (432.61-645.83) | -0.98 (-2.39 - 0.45) | 6185 (4360-8094) | 14058 (9118-21639) | 186.01 (131.14-243.42) | 255.54 (165.74-393.34) | 1.16 (0.36 - 1.97) |
| Honduras | 19961 (15974-25162) | 29506 (23304-37932) | 736.28 (589.24-928.13) | 676.29 (534.14-869.43) | -0.65 (-1.28 - -0.02) | 4188 (2984-5551) | 4527 (3119-6224) | 154.46 (110.09-204.75) | 103.75 (71.49-142.65) | -1.34 (-1.57 - -1.11) |
| Hungary | 24156 (18958-31309) | 15286 (11916-19798) | 833.26 (653.95-1079.99) | 817.35 (637.14-1058.59) | -0.05 (-0.11 - 0.01) | 2112 (1352-3181) | 978 (572-1583) | 72.86 (46.62-109.73) | 52.32 (30.61-84.66) | -1.13 (-1.31 - -0.94) |
| Iceland | 441 (344-570) | 424 (322-562) | 521.74 (407.39-673.91) | 478.6 (364.05-635.42) | -0.26 (-0.39 - -0.14) | 31 (18-50) | 29 (17-48) | 36.18 (21.03-58.88) | 32.86 (18.94-53.83) | -0.33 (-0.41 - -0.25) |
| India | 1110060 (892956-1391089) | 1116563 (884234-1393840) | 267.65 (215.31-335.42) | 218.72 (173.21-273.03) | -0.6 (-1.16 - -0.04) | 181225 (133023-235524) | 151237 (109371-202001) | 43.7 (32.07-56.79) | 29.62 (21.42-39.57) | -1.26 (-1.56 - -0.96) |
| Indonesia | 202006 (150310-274488) | 194503 (144722-262667) | 229.94 (171.1-312.45) | 221.01 (164.45-298.46) | -0.06 (-0.86 - 0.75) | 21685 (14584-31022) | 19544 (12643-29050) | 24.68 (16.6-35.31) | 22.21 (14.37-33.01) | -0.46 (-1 - 0.08) |
| Iran (Islamic Republic of) | 280429 (210608-365664) | 64163 (49940-81884) | 874.5 (656.77-1140.3) | 248.12 (193.12-316.65) | -1.69 (-2.62 - -0.75) | 23965 (14874-37573) | 4061 (2576-6462) | 74.73 (46.38-117.17) | 15.7 (9.96-24.99) | -5.06 (-5.74 - -4.38) |
| Iraq | 49970 (38075-65862) | 69115 (54634-89847) | 498.44 (379.78-656.95) | 379.95 (300.34-493.92) | -1.15 (-7.39 - 5.51) | 23586 (10442-51212) | 12725 (6898-23172) | 235.26 (104.15-510.82) | 69.95 (37.92-127.39) | -4.13 (-5.23 - -3.02) |
| Ireland | 7999 (6238-10373) | 7043 (5367-9447) | 602.3 (469.71-781.11) | 525.93 (400.74-705.41) | -0.47 (-0.53 - -0.4) | 638 (360-1034) | 496 (284-811) | 48.05 (27.12-77.84) | 37.07 (21.17-60.57) | -0.91 (-0.95 - -0.86) |
| Israel | 11721 (9236-15264) | 16487 (12689-21880) | 584.42 (460.52-761.11) | 497.53 (382.92-660.26) | -0.46 (-0.78 - -0.13) | 865 (518-1368) | 1116 (665-1766) | 43.13 (25.85-68.22) | 33.69 (20.07-53.29) | -0.81 (-1.25 - -0.38) |
| Italy | 71669 (53149-95596) | 35531 (26543-47600) | 526.2 (390.22-701.87) | 327.86 (244.93-439.23) | -1.66 (-1.91 - -1.41) | 5208 (3027-8697) | 2446 (1442-3962) | 38.24 (22.23-63.85) | 22.57 (13.31-36.56) | -1.84 (-1.97 - -1.72) |
| Jamaica | 6933 (5497-8794) | 5312 (4074-6953) | 635.63 (503.92-806.2) | 615.01 (471.69-805.01) | -0.07 (-0.31 - 0.16) | 821 (564-1161) | 553 (364-806) | 75.23 (51.71-106.45) | 64.06 (42.15-93.3) | -0.52 (-0.6 - -0.44) |
| Japan | 177094 (139263-226224) | 106407 (83177-136335) | 530.99 (417.56-678.3) | 492.69 (385.13-631.27) | -0.24 (-0.48 - 0) | 11916 (6717-19948) | 6698 (3787-11180) | 35.73 (20.14-59.81) | 31.01 (17.53-51.77) | -0.47 (-0.52 - -0.42) |
| Jordan | 6216 (4880-7952) | 12977 (9933-16929) | 291.52 (228.86-372.93) | 264.22 (202.24-344.7) | -0.4 (-0.64 - -0.16) | 647 (442-913) | 852 (529-1354) | 30.33 (20.75-42.81) | 17.35 (10.77-27.57) | -1.83 (-2 - -1.67) |
| Kazakhstan | 42866 (34549-53081) | 34926 (27782-44002) | 647.93 (522.22-802.33) | 557.7 (443.62-702.63) | -0.49 (-0.64 - -0.35) | 4573 (3207-6370) | 2268 (1460-3389) | 69.12 (48.47-96.29) | 36.21 (23.31-54.11) | -2.22 (-2.32 - -2.13) |
| Kenya | 40333 (31919-50990) | 72948 (57133-92429) | 291.35 (230.57-368.33) | 291.52 (228.32-369.37) | 0.15 (-0.24 - 0.55) | 7007 (5076-9121) | 12271 (8921-16163) | 50.62 (36.67-65.89) | 49.04 (35.65-64.59) | -0.08 (-0.21 - 0.06) |
| Kiribati | 64 (50-81) | 105 (82-135) | 175.66 (139.06-224.34) | 197.73 (155.73-255.81) | 0.13 (-0.51 - 0.78) | 13 (9-18) | 20 (14-27) | 36.25 (25.05-48.87) | 37.97 (26.87-50.72) | 0.15 (-0.04 - 0.34) |
| Kuwait | 6033 (3356-11731) | 3401 (2539-4542) | 863.77 (480.5-1679.55) | 310.17 (231.55-414.24) | -4.44 (-5.72 - -3.15) | 199 (123-298) | 197 (115-331) | 28.48 (17.56-42.7) | 17.95 (10.45-30.18) | -1.59 (-2.03 - -1.15) |
| Kyrgyzstan | 12529 (10118-15638) | 14108 (11089-17883) | 596.99 (482.09-745.12) | 539.78 (424.27-684.2) | -0.26 (-0.7 - 0.19) | 1456 (1026-1983) | 1159 (783-1689) | 69.39 (48.89-94.48) | 44.33 (29.97-64.63) | -1.48 (-1.65 - -1.3) |
| Lao People's Democratic Republic | 7588 (5950-10415) | 6158 (4983-7720) | 336.99 (264.25-462.53) | 208.27 (168.53-261.1) | -0.96 (-1.4 - -0.52) | 1508 (1077-1976) | 1021 (714-1381) | 66.98 (47.83-87.75) | 34.53 (24.15-46.7) | -2.26 (-2.37 - -2.15) |
| Latvia | 5982 (4897-7392) | 2522 (1987-3222) | 795.03 (650.79-982.32) | 643.26 (506.8-821.7) | -0.72 (-0.76 - -0.68) | 498 (329-730) | 147 (86-241) | 66.21 (43.75-97.08) | 37.46 (21.88-61.59) | -1.93 (-2.05 - -1.82) |
| Lebanon | 8503 (5839-13718) | 5176 (3932-6778) | 545.82 (374.85-880.63) | 299.57 (227.58-392.32) | -0.89 (-1.6 - -0.17) | 2225 (843-5672) | 296 (178-483) | 142.85 (54.1-364.09) | 17.15 (10.29-27.93) | -6.97 (-7.51 - -6.42) |
| Lesotho | 1879 (1526-2353) | 1673 (1329-2115) | 195.73 (158.95-245.09) | 189.96 (150.95-240.17) | 0.03 (-0.12 - 0.18) | 186 (122-262) | 164 (105-240) | 19.35 (12.66-27.32) | 18.64 (11.98-27.21) | -0.08 (-0.2 - 0.03) |
| Liberia | 13930 (5323-33572) | 3332 (2543-4379) | 1262.68 (482.47-3043.22) | 138.96 (106.05-182.61) | -4.27 (-7.28 - -1.17) | 371 (206-622) | 367 (243-543) | 33.59 (18.67-56.38) | 15.32 (10.12-22.66) | -2.63 (-4.05 - -1.19) |
| Libya | 6476 (5052-8284) | 8354 (6023-12543) | 274.29 (213.95-350.83) | 406.25 (292.88-609.99) | 1.71 (0.04 - 3.41) | 889 (592-1287) | 779 (494-1207) | 37.64 (25.07-54.51) | 37.9 (24.02-58.71) | 0.3 (-0.53 - 1.13) |
| Lithuania | 8082 (6482-10236) | 3722 (2926-4804) | 730.92 (586.22-925.69) | 671.89 (528.09-867.12) | -0.22 (-0.37 - -0.08) | 630 (407-955) | 225 (133-367) | 56.99 (36.85-86.38) | 40.57 (23.95-66.24) | -1.08 (-1.39 - -0.77) |
| Luxembourg | 552 (425-721) | 667 (512-883) | 625.58 (482.06-817.08) | 504.31 (387.02-667.04) | -0.73 (-0.84 - -0.62) | 37 (21-61) | 46 (27-76) | 42.01 (24.09-69.6) | 34.52 (20.17-57.44) | -0.67 (-0.73 - -0.6) |
| Madagascar | 23918 (18561-31340) | 42136 (30889-56800) | 353.06 (273.98-462.61) | 303.75 (222.68-409.46) | -0.56 (-0.72 - -0.39) | 1925 (1291-2828) | 3491 (2296-5070) | 28.42 (19.06-41.75) | 25.17 (16.56-36.55) | -0.41 (-0.5 - -0.32) |
| Malawi | 15614 (11757-20586) | 29191 (21049-40394) | 287.93 (216.81-379.63) | 286.92 (206.89-397.03) | -0.03 (-0.32 - 0.27) | 1264 (835-1818) | 2327 (1533-3448) | 23.32 (15.4-33.53) | 22.87 (15.06-33.89) | -0.07 (-0.21 - 0.07) |
| Malaysia | 21488 (17091-27343) | 26151 (20704-33541) | 257.53 (204.84-327.71) | 250.94 (198.67-321.85) | -0.03 (-0.19 - 0.13) | 3016 (2080-4061) | 2232 (1402-3373) | 36.15 (24.92-48.67) | 21.41 (13.46-32.37) | -1.77 (-1.89 - -1.65) |
| Maldives | 357 (288-448) | 338 (267-431) | 279.16 (225.18-349.9) | 241.06 (190.54-307.17) | -0.79 (-1.32 - -0.25) | 53 (37-71) | 24 (14-37) | 41.02 (28.71-55.5) | 16.8 (10.29-26.34) | -3.1 (-3.63 - -2.57) |
| Mali | 10250 (8040-13631) | 22808 (17939-29450) | 207.61 (162.85-276.09) | 178.52 (140.41-230.51) | -0.55 (-6.49 - 5.78) | 828 (565-1165) | 2091 (1349-3090) | 16.77 (11.44-23.59) | 16.37 (10.56-24.18) | -0.09 (-0.73 - 0.55) |
| Malta | 660 (514-856) | 464 (357-613) | 572.93 (445.87-743.01) | 555.32 (427.23-733.68) | -0.09 (-0.18 - -0.01) | 47 (27-76) | 31 (18-49) | 40.46 (23.77-65.53) | 37.14 (21.59-58.74) | -0.26 (-0.34 - -0.18) |
| Marshall Islands | 53 (42-67) | 48 (39-61) | 194.75 (155.5-248.79) | 202.27 (161.34-254.17) | 0.14 (0.03 - 0.25) | 9 (6-12) | 8 (5-11) | 34.65 (24.02-45.91) | 33.12 (22.95-44.27) | -0.14 (-0.25 - -0.02) |
| Mauritania | 1925 (1566-2401) | 3179 (2501-4151) | 168.1 (136.71-209.61) | 154.16 (121.29-201.28) | -0.22 (-0.66 - 0.21) | 193 (128-274) | 290 (189-422) | 16.82 (11.21-23.91) | 14.07 (9.14-20.47) | -0.56 (-0.68 - -0.43) |
| Mauritius | 1601 (1295-1990) | 717 (539-941) | 370.75 (299.83-460.9) | 232.54 (174.92-305.48) | -1.65 (-1.89 - -1.41) | 97 (56-155) | 48 (27-79) | 22.46 (12.99-35.93) | 15.5 (8.81-25.51) | -1.23 (-1.36 - -1.1) |
| Mexico | 294645 (224459-398092) | 231462 (167023-326017) | 678.24 (516.68-916.37) | 535.57 (386.47-754.36) | -0.7 (-1.76 - 0.36) | 23928 (14442-37586) | 15686 (8550-26099) | 55.08 (33.24-86.52) | 36.3 (19.78-60.39) | -1.24 (-2.61 - 0.14) |
| Micronesia (Federated States of) | 115 (92-145) | 89 (70-114) | 196.74 (157.21-246.72) | 210.06 (165.04-268) | 0.28 (-0.29 - 0.86) | 22 (16-30) | 14 (10-19) | 38.13 (27.05-50.63) | 33.04 (22.88-44.68) | -0.3 (-0.84 - 0.26) |
| Monaco | 16 (13-21) | 24 (18-31) | 336.19 (258.84-435.12) | 348.77 (267.58-459.15) | 0.14 (0.1 - 0.18) | 1 (1-2) | 1 (1-2) | 21.1 (11.58-35.43) | 22.07 (12.16-36.34) | 0.17 (0.08 - 0.26) |
| Mongolia | 6360 (5221-7883) | 6968 (5582-8765) | 561.54 (460.96-696.03) | 565.98 (453.4-711.89) | 0.05 (-0.46 - 0.57) | 1002 (709-1314) | 668 (459-941) | 88.48 (62.57-116.01) | 54.22 (37.26-76.4) | -1.68 (-1.73 - -1.62) |
| Montenegro | 1826 (1450-2348) | 1221 (958-1578) | 850.78 (675.67-1094.4) | 815.8 (640.26-1054.7) | -0.12 (-0.23 - -0.01) | 130 (81-204) | 78 (46-127) | 60.69 (37.56-94.85) | 52.27 (30.78-85) | -0.5 (-0.58 - -0.42) |
| Morocco | 44743 (34474-57435) | 35672 (27618-46028) | 358.11 (275.92-459.69) | 284.39 (220.18-366.95) | -1.05 (-1.23 - -0.88) | 6221 (4433-8341) | 3121 (2106-4519) | 49.79 (35.48-66.76) | 24.88 (16.79-36.03) | -2.36 (-2.45 - -2.26) |
| Mozambique | 33712 (25315-46648) | 56903 (43621-74689) | 453.93 (340.85-628.1) | 334.47 (256.4-439.01) | -1.25 (-2.17 - -0.32) | 8329 (3701-19110) | 4284 (2895-6373) | 112.15 (49.83-257.31) | 25.18 (17.02-37.46) | -5.04 (-5.5 - -4.57) |
| Myanmar | 69487 (54577-89498) | 55495 (43826-70677) | 348.53 (273.74-448.9) | 278.31 (219.79-354.45) | -0.9 (-1.78 - -0.02) | 13990 (9386-22335) | 11587 (7797-16670) | 70.17 (47.08-112.02) | 58.11 (39.1-83.6) | -0.84 (-1.37 - -0.3) |
| Namibia | 1459 (1164-1835) | 2044 (1588-2596) | 193.06 (153.98-242.73) | 189.68 (147.3-240.86) | -0.08 (-0.31 - 0.16) | 327 (181-626) | 168 (106-251) | 43.22 (23.93-82.8) | 15.6 (9.79-23.33) | -3.41 (-3.68 - -3.13) |
| Nauru | 9 (7-12) | 9 (7-12) | 171.42 (131.25-225.87) | 185.72 (140.26-245.69) | 0.3 (0.08 - 0.54) | 1 (0-1) | 1 (0-1) | 11.46 (7.04-17.35) | 12.22 (7.36-19.02) | 0.25 (0.14 - 0.37) |
| Nepal | 22823 (17713-29452) | 27881 (21848-35696) | 218.95 (169.93-282.54) | 222.71 (174.51-285.13) | -0.14 (-0.88 - 0.62) | 4085 (2873-5363) | 5388 (3728-7465) | 39.19 (27.56-51.45) | 43.04 (29.78-59.63) | 0.41 (-0.18 - 1) |
| Netherlands | 17534 (13658-22461) | 16903 (13041-21855) | 457.51 (356.37-586.06) | 448.36 (345.92-579.74) | -0.09 (-0.23 - 0.05) | 1240 (728-2039) | 1199 (695-1961) | 32.35 (19-53.19) | 31.82 (18.43-52.02) | -0.06 (-0.18 - 0.05) |
| New Zealand | 13425 (10361-17492) | 12234 (9578-15766) | 1219.09 (940.85-1588.36) | 1033.1 (808.78-1331.3) | -0.58 (-0.65 - -0.5) | 924 (566-1478) | 739 (448-1203) | 83.9 (51.41-134.21) | 62.4 (37.86-101.55) | -1.02 (-1.07 - -0.97) |
| Nicaragua | 16047 (12682-20339) | 16390 (12744-21410) | 711.9 (562.62-902.33) | 623.45 (484.76-814.41) | -0.74 (-1.13 - -0.36) | 5772 (3302-10668) | 1640 (1082-2410) | 256.05 (146.47-473.29) | 62.37 (41.17-91.68) | -4.76 (-4.89 - -4.63) |
| Niger | 8948 (7248-11142) | 22684 (18173-28785) | 184.82 (149.69-230.13) | 155.82 (124.84-197.73) | -0.31 (-0.59 - -0.03) | 835 (547-1195) | 2090 (1394-2964) | 17.25 (11.29-24.68) | 14.36 (9.58-20.36) | -0.63 (-0.82 - -0.45) |
| Nigeria | 78355 (62445-99985) | 182780 (145141-233379) | 160.74 (128.1-205.11) | 155.15 (123.2-198.1) | -0.06 (-1.06 - 0.96) | 7412 (5050-10454) | 18540 (12283-26745) | 15.2 (10.36-21.45) | 15.74 (10.43-22.7) | 0.15 (0 - 0.3) |
| Niue | 2 (1-3) | 1 (1-2) | 192.41 (141.87-260.25) | 214.31 (156.47-292.27) | 0.33 (-0.52 - 1.18) | 0 (0-0) | 0 (0-0) | 12.3 (7.21-19.58) | 14.93 (9-23.24) | 0.41 (-0.9 - 1.75) |
| North Macedonia | 7591 (5871-9983) | 3639 (2856-4669) | 1072.76 (829.66-1410.66) | 778.37 (610.86-998.64) | -1.06 (-1.26 - -0.86) | 652 (431-952) | 228 (133-373) | 92.14 (60.93-134.49) | 48.7 (28.44-79.7) | -2.15 (-2.4 - -1.9) |
| Northern Mariana Islands | 36 (27-48) | 30 (22-41) | 219.77 (165.4-293.51) | 259.56 (186.92-351.86) | 1.24 (0.48 - 2.01) | 2 (1-4) | 2 (1-3) | 13.58 (7.86-21.95) | 17.4 (9.67-28.95) | 0.94 (0.36 - 1.52) |
| Norway | 5218 (4138-6578) | 5132 (3965-6683) | 468.66 (371.65-590.81) | 408.75 (315.82-532.27) | -0.47 (-0.68 - -0.26) | 317 (175-531) | 318 (175-529) | 28.43 (15.69-47.72) | 25.32 (13.97-42.16) | -0.36 (-0.49 - -0.23) |
| Oman | 2849 (2165-3768) | 3735 (2811-4967) | 286.54 (217.74-378.97) | 291.16 (219.13-387.23) | 0.08 (-0.1 - 0.25) | 270 (186-387) | 207 (123-342) | 27.2 (18.72-38.93) | 16.11 (9.59-26.69) | -1.74 (-1.97 - -1.51) |
| Pakistan | 102326 (81417-129753) | 190391 (151573-241326) | 163.6 (130.17-207.45) | 170.19 (135.49-215.73) | 0.43 (-0.26 - 1.11) | 17588 (12596-23160) | 32620 (23296-44009) | 28.12 (20.14-37.03) | 29.16 (20.82-39.34) | 0.15 (-0.28 - 0.58) |
| Palau | 18 (14-23) | 12 (9-15) | 284.3 (224.09-354.49) | 263.92 (205.78-339.15) | -0.24 (-0.31 - -0.17) | 1 (1-2) | 1 (0-1) | 18.06 (10.58-28.75) | 15.33 (8.75-25.12) | -0.56 (-0.66 - -0.46) |
| Palestine | 6119 (4033-10347) | 7412 (5701-9671) | 501.33 (330.4-847.73) | 312.1 (240.07-407.24) | -1.1 (-11.16 - 10.11) | 1391 (632-3079) | 1257 (722-2180) | 113.93 (51.81-252.24) | 52.91 (30.4-91.81) | -2.56 (-3.3 - -1.82) |
| Panama | 7270 (5734-9259) | 9772 (7651-12654) | 669.01 (527.67-851.99) | 654.12 (512.18-847.05) | -0.08 (-0.2 - 0.03) | 1063 (737-1504) | 835 (539-1247) | 97.86 (67.81-138.42) | 55.92 (36.08-83.47) | -1.88 (-1.98 - -1.78) |
| Papua New Guinea | 5395 (4385-6728) | 12104 (9791-15045) | 255.83 (207.95-319.02) | 261.12 (211.2-324.56) | -0.21 (-0.78 - 0.36) | 1132 (778-1506) | 2189 (1557-2928) | 53.7 (36.9-71.41) | 47.22 (33.59-63.17) | -0.71 (-1.3 - -0.13) |
| Paraguay | 9300 (7365-11814) | 10727 (8454-13640) | 448.63 (355.29-569.92) | 410.1 (323.23-521.51) | -0.29 (-0.4 - -0.18) | 1210 (855-1638) | 984 (658-1418) | 58.38 (41.27-79.01) | 37.64 (25.16-54.21) | -1.46 (-1.55 - -1.37) |
| Peru | 66159 (53057-85442) | 55919 (44164-71317) | 615.63 (493.72-795.07) | 466.06 (368.08-594.4) | -1.01 (-1.35 - -0.68) | 10649 (7495-14672) | 4694 (3057-6946) | 99.09 (69.75-136.53) | 39.12 (25.48-57.89) | -3.12 (-3.33 - -2.92) |
| Philippines | 98403 (79519-123533) | 97222 (77766-122645) | 304.89 (246.38-382.76) | 209.95 (167.93-264.84) | -1.66 (-3.11 - -0.19) | 13759 (9768-18846) | 12871 (9340-17302) | 42.63 (30.27-58.39) | 27.79 (20.17-37.36) | -1.55 (-1.68 - -1.43) |
| Poland | 110532 (86569-143572) | 61218 (47575-79329) | 889.49 (696.65-1155.38) | 794.21 (617.21-1029.18) | -0.37 (-0.42 - -0.33) | 10224 (6816-14972) | 3710 (2178-6118) | 82.28 (54.85-120.48) | 48.13 (28.26-79.37) | -1.81 (-1.98 - -1.63) |
| Portugal | 14314 (11423-18268) | 7611 (5888-9946) | 480.4 (383.38-613.09) | 386.29 (298.87-504.83) | -0.74 (-0.85 - -0.63) | 1300 (826-2003) | 555 (319-921) | 43.65 (27.71-67.22) | 28.18 (16.19-46.76) | -1.48 (-1.64 - -1.31) |
| Puerto Rico | 7933 (5994-10543) | 4796 (3463-6580) | 595.4 (449.86-791.34) | 635.6 (458.98-871.97) | 0.32 (0.13 - 0.5) | 533 (301-874) | 320 (171-556) | 40.04 (22.61-65.58) | 42.43 (22.62-73.72) | 0.37 (0.02 - 0.73) |
| Qatar | 500 (390-645) | 1610 (1217-2142) | 327.59 (255.56-422.14) | 311.07 (235.11-413.72) | -0.16 (-0.3 - -0.03) | 47 (32-66) | 91 (54-152) | 30.62 (20.98-43.38) | 17.56 (10.45-29.37) | -1.89 (-2.01 - -1.77) |
| Republic of Korea | 96042 (75952-123642) | 55919 (43671-72014) | 600.41 (474.81-772.95) | 586.63 (458.14-755.48) | -0.07 (-0.21 - 0.07) | 9470 (6279-13807) | 3729 (2172-6133) | 59.2 (39.26-86.31) | 39.12 (22.78-64.34) | -1.39 (-1.51 - -1.26) |
| Republic of Moldova | 11645 (9334-14615) | 4818 (3806-6081) | 738.92 (592.27-927.32) | 623.4 (492.48-786.84) | -0.5 (-0.73 - -0.28) | 1110 (759-1576) | 355 (225-550) | 70.42 (48.17-99.99) | 45.89 (29.18-71.2) | -1.42 (-1.58 - -1.26) |
| Romania | 66447 (53592-83480) | 31503 (24751-40534) | 885.36 (714.08-1112.32) | 776.39 (609.99-998.98) | -0.45 (-0.5 - -0.39) | 6777 (4649-9660) | 1979 (1168-3201) | 90.29 (61.94-128.71) | 48.76 (28.78-78.88) | -2.08 (-2.26 - -1.9) |
| Russian Federation | 351772 (277874-440648) | 187798 (146055-244374) | 777.34 (614.04-973.74) | 546.21 (424.8-710.76) | -1.12 (-1.47 - -0.77) | 28532 (18957-41575) | 10660 (6398-17371) | 63.05 (41.89-91.87) | 31 (18.61-50.52) | -2.36 (-2.67 - -2.05) |
| Rwanda | 21690 (14389-35572) | 18060 (13229-24579) | 525.75 (348.78-862.24) | 288.95 (211.65-393.25) | -1.73 (-2.79 - -0.66) | 1281 (865-1843) | 1487 (953-2197) | 31.05 (20.96-44.68) | 23.79 (15.25-35.16) | -0.87 (-2.51 - 0.8) |
| Saint Kitts and Nevis | 133 (104-167) | 104 (82-133) | 724.93 (566.44-912.01) | 638.47 (504.04-815.34) | -0.46 (-0.72 - -0.2) | 8 (5-13) | 6 (4-11) | 45.01 (25.61-73.18) | 39.1 (21.54-65.94) | -0.46 (-0.56 - -0.36) |
| Saint Lucia | 395 (318-495) | 268 (212-342) | 584.4 (469.93-732.32) | 591.64 (468.15-756.12) | 0.03 (-0.08 - 0.14) | 58 (41-78) | 30 (20-43) | 86.11 (60.48-115.71) | 65.89 (43.71-95.43) | -0.9 (-1.06 - -0.74) |
| Saint Vincent and the Grenadines | 339 (271-426) | 197 (157-250) | 624.83 (500.63-784.86) | 578.04 (460.19-730.63) | -0.36 (-0.5 - -0.22) | 47 (33-64) | 24 (17-34) | 86.89 (61.57-117.9) | 70.29 (49.19-98.76) | -0.71 (-0.84 - -0.59) |
| Samoa | 237 (188-302) | 226 (174-291) | 275.53 (218.45-350.8) | 231.75 (178.95-298.99) | 0.07 (-0.86 - 1) | 29 (20-39) | 40 (28-56) | 33.57 (23.24-45.05) | 41.08 (28.25-57.12) | 0.85 (-0.29 - 2) |
| San Marino | 22 (17-29) | 26 (20-34) | 335.88 (258.16-442.77) | 347.8 (265.62-457.03) | 0.14 (0.09 - 0.2) | 1 (1-2) | 2 (1-3) | 22.29 (12.2-37.15) | 22.48 (12.1-37.51) | 0.04 (-0.01 - 0.08) |
| Sao Tome and Principe | 118 (90-154) | 167 (127-224) | 167.64 (128.88-219.13) | 172.03 (130.1-230.64) | 0.11 (0.05 - 0.17) | 12 (8-17) | 15 (10-22) | 17.19 (11.58-24.72) | 15.38 (9.78-22.61) | -0.38 (-0.46 - -0.29) |
| Saudi Arabia | 38579 (28918-50664) | 35524 (27335-46274) | 462.37 (346.58-607.21) | 367.3 (282.63-478.44) | -0.84 (-0.96 - -0.71) | 3408 (2311-4962) | 1969 (1170-3278) | 40.85 (27.7-59.47) | 20.36 (12.1-33.89) | -2.38 (-2.44 - -2.33) |
| Senegal | 7407 (6007-9268) | 11538 (8973-14837) | 166.99 (135.43-208.94) | 148.34 (115.36-190.75) | -0.39 (-0.9 - 0.13) | 693 (462-997) | 1109 (738-1635) | 15.62 (10.41-22.47) | 14.26 (9.48-21.01) | -0.24 (-0.47 - -0.02) |
| Serbia | 23064 (18207-29438) | 15927 (12379-20505) | 819.66 (647.04-1046.17) | 799.98 (621.74-1029.88) | -0.35 (-0.74 - 0.03) | 2180 (1443-3141) | 1014 (596-1637) | 77.48 (51.28-111.63) | 50.93 (29.95-82.23) | -1.57 (-1.97 - -1.16) |
| Seychelles | 75 (58-96) | 59 (45-78) | 238.42 (183.64-305.11) | 205.67 (156.22-269.73) | -0.73 (-1.07 - -0.39) | 6 (3-9) | 4 (2-6) | 18.46 (11.01-28.41) | 13.55 (7.77-21.78) | -0.96 (-1.19 - -0.72) |
| Sierra Leone | 3157 (2540-3969) | 5898 (4579-7584) | 162.64 (130.84-204.48) | 138.14 (107.25-177.64) | -0.78 (-13.37 - 13.64) | 304 (203-437) | 804 (525-1165) | 15.67 (10.47-22.5) | 18.83 (12.31-27.28) | 0.87 (0.28 - 1.46) |
| Singapore | 5190 (4034-6737) | 5671 (4437-7266) | 563.55 (438.02-731.46) | 547.39 (428.29-701.39) | -0.09 (-0.19 - 0.02) | 389 (235-612) | 355 (207-583) | 42.19 (25.56-66.49) | 34.24 (20.02-56.3) | -0.65 (-0.9 - -0.41) |
| Slovakia | 14182 (11164-18121) | 8878 (6943-11336) | 808.3 (636.25-1032.77) | 796.95 (623.29-1017.66) | -0.04 (-0.2 - 0.13) | 1123 (715-1691) | 540 (318-875) | 63.99 (40.76-96.38) | 48.5 (28.55-78.51) | -0.94 (-1.03 - -0.84) |
| Slovenia | 4937 (3880-6281) | 3676 (2866-4735) | 887.06 (697.02-1128.5) | 912.5 (711.51-1175.23) | 0.12 (-0.08 - 0.31) | 338 (203-540) | 228 (134-374) | 60.79 (36.52-97.05) | 56.55 (33.36-92.78) | -0.22 (-0.38 - -0.07) |
| Solomon Islands | 424 (339-537) | 706 (566-893) | 216.4 (172.98-273.93) | 216.79 (173.79-274.04) | 0.2 (-0.25 - 0.65) | 82 (58-110) | 106 (75-141) | 41.99 (29.74-55.99) | 32.41 (23.13-43.39) | -0.82 (-1.1 - -0.55) |
| Somalia | 22096 (14527-36842) | 37607 (28754-49707) | 518.96 (341.18-865.28) | 318.71 (243.68-421.26) | 0.28 (-0.52 - 1.08) | 2006 (986-5362) | 4456 (2700-7602) | 47.12 (23.15-125.94) | 37.76 (22.88-64.43) | -0.87 (-2.17 - 0.44) |
| South Africa | 40367 (30654-52669) | 34510 (25652-46540) | 233.75 (177.51-304.99) | 176.21 (130.98-237.64) | -0.94 (-1.13 - -0.74) | 3615 (2383-5227) | 2722 (1755-3995) | 20.93 (13.8-30.27) | 13.9 (8.96-20.4) | -1.35 (-1.57 - -1.13) |
| South Sudan | 10833 (8279-14292) | 17988 (13816-23775) | 330.66 (252.69-436.25) | 339.65 (260.87-448.91) | 0.76 (-5.36 - 7.27) | 1453 (898-2383) | 2266 (1353-3707) | 44.36 (27.42-72.75) | 42.78 (25.56-69.99) | 0.03 (-0.7 - 0.76) |
| Spain | 52363 (40842-68093) | 40327 (30590-54569) | 469.8 (366.43-610.92) | 446.16 (338.43-603.72) | -0.15 (-0.36 - 0.06) | 3831 (2234-6266) | 2806 (1645-4550) | 34.37 (20.04-56.22) | 31.05 (18.2-50.34) | -0.35 (-0.43 - -0.28) |
| Sri Lanka | 48217 (30423-86150) | 18743 (15068-23674) | 659.32 (416-1178) | 274.58 (220.74-346.82) | -2.37 (-3.5 - -1.21) | 4165 (2728-6173) | 2068 (1328-2976) | 56.95 (37.31-84.41) | 30.29 (19.46-43.6) | -2.42 (-3.06 - -1.78) |
| Sudan | 36044 (23194-61334) | 28983 (23163-36126) | 321.03 (206.58-546.29) | 143.1 (114.37-178.37) | -1.49 (-2.03 - -0.94) | 2680 (1619-4271) | 2859 (1817-4457) | 23.87 (14.42-38.04) | 14.12 (8.97-22.01) | -1.78 (-2.44 - -1.13) |
| Suriname | 944 (761-1174) | 1058 (842-1328) | 553.78 (446.13-688.57) | 546.05 (434.59-685.71) | -0.01 (-0.08 - 0.07) | 170 (123-226) | 137 (96-189) | 99.9 (72.04-132.7) | 70.84 (49.81-97.53) | -1.16 (-1.25 - -1.08) |
| Sweden | 12099 (9502-15296) | 11195 (8732-14587) | 574.13 (450.89-725.8) | 472.39 (368.46-615.52) | -0.66 (-0.78 - -0.53) | 782 (416-1350) | 703 (374-1178) | 37.09 (19.72-64.08) | 29.67 (15.79-49.72) | -0.76 (-0.85 - -0.66) |
| Switzerland | 9218 (7183-12110) | 9203 (6939-12323) | 585.05 (455.88-768.62) | 528.31 (398.32-707.38) | -0.34 (-0.51 - -0.17) | 613 (357-988) | 614 (357-990) | 38.92 (22.68-62.68) | 35.22 (20.52-56.85) | -0.31 (-0.42 - -0.19) |
| Syrian Arab Republic | 20573 (16067-26285) | 21306 (15939-28946) | 273.56 (213.63-349.5) | 362.03 (270.83-491.85) | 1.15 (-7.23 - 10.28) | 3126 (1930-5586) | 6463 (3299-12224) | 41.56 (25.67-74.27) | 109.82 (56.06-207.71) | 3.26 (2.1 - 4.44) |
| Taiwan (Province of China) | 12473 (10104-15481) | 5225 (4115-6658) | 170.47 (138.09-211.57) | 122.35 (96.36-155.91) | -1.68 (-2.15 - -1.2) | 995 (620-1489) | 378 (226-606) | 13.59 (8.47-20.35) | 8.84 (5.3-14.19) | -1.46 (-1.82 - -1.1) |
| Tajikistan | 18102 (14729-22383) | 21166 (16611-26905) | 631.22 (513.6-780.49) | 514 (403.37-653.35) | -1.34 (-1.93 - -0.75) | 2255 (1614-3047) | 2272 (1620-3174) | 78.62 (56.29-106.23) | 55.17 (39.35-77.08) | -0.87 (-1.83 - 0.1) |
| Thailand | 66844 (53070-85000) | 35370 (28183-45391) | 292.01 (231.84-371.33) | 236.46 (188.42-303.46) | -0.8 (-1.11 - -0.48) | 9223 (6407-12928) | 2900 (1796-4490) | 40.29 (27.99-56.47) | 19.39 (12.01-30.02) | -2.49 (-2.74 - -2.24) |
| Timor-Leste | 2099 (1363-3661) | 1408 (1127-1770) | 513.72 (333.56-896.03) | 211.64 (169.49-266.12) | -2.99 (-4.97 - -0.97) | 634 (319-1281) | 215 (151-290) | 155.08 (78.14-313.42) | 32.4 (22.7-43.61) | -5.41 (-6.04 - -4.78) |
| Togo | 3557 (2877-4505) | 6046 (4713-7822) | 164.11 (132.74-207.84) | 152.82 (119.11-197.71) | -0.42 (-0.66 - -0.18) | 342 (226-487) | 573 (388-822) | 15.78 (10.44-22.49) | 14.49 (9.8-20.79) | -0.23 (-0.58 - 0.13) |
| Tokelau | 2 (1-2) | 1 (1-2) | 195.02 (147.24-261.39) | 234.08 (169.27-324.16) | 0.66 (0.49 - 0.84) | 0 (0-0) | 0 (0-0) | 14.07 (8.73-21.2) | 14.71 (8.67-23.82) | 0.25 (-0.02 - 0.53) |
| Tonga | 128 (105-158) | 109 (87-137) | 249.7 (204-308.28) | 234.16 (187.83-294.4) | -0.08 (-0.4 - 0.24) | 19 (13-26) | 14 (9-19) | 36.94 (25.76-49.77) | 29.31 (20.18-40.15) | -0.77 (-1.04 - -0.5) |
| Trinidad and Tobago | 2974 (2383-3746) | 1978 (1565-2520) | 574.85 (460.73-724.2) | 547.11 (432.97-697.19) | -0.13 (-0.33 - 0.08) | 433 (305-583) | 210 (141-303) | 83.78 (59.03-112.7) | 58.03 (39.06-83.91) | -1.22 (-1.36 - -1.09) |
| Tunisia | 11143 (8740-14344) | 8836 (6762-11585) | 273.5 (214.52-352.06) | 255.99 (195.91-335.61) | -0.18 (-0.36 - 0.01) | 1093 (744-1546) | 528 (323-864) | 26.82 (18.25-37.96) | 15.31 (9.36-25.04) | -1.86 (-1.98 - -1.73) |
| Turkey | 86007 (67364-111781) | 61594 (47315-81272) | 306.96 (240.43-398.95) | 276.27 (212.23-364.54) | -0.72 (-1.35 - -0.08) | 9820 (6880-13517) | 3944 (2351-6376) | 35.05 (24.56-48.24) | 17.69 (10.54-28.6) | -2.4 (-2.89 - -1.91) |
| Turkmenistan | 13512 (11156-16557) | 11506 (9214-14373) | 715.83 (591-877.13) | 600.59 (480.97-750.24) | -0.69 (-0.96 - -0.42) | 1529 (1083-2083) | 915 (617-1338) | 81 (57.36-110.33) | 47.75 (32.21-69.83) | -1.8 (-1.99 - -1.62) |
| Tuvalu | 7 (5-9) | 9 (6-12) | 166.84 (127.39-218.92) | 196.66 (141.83-269.84) | 0.61 (0.38 - 0.83) | 1 (0-1) | 1 (0-1) | 12.63 (8.06-18.64) | 14.22 (8.52-22.07) | 0.43 (0.23 - 0.63) |
| Uganda | 31734 (24296-42146) | 67382 (48895-90947) | 307.61 (235.51-408.54) | 281.72 (204.43-380.25) | -0.4 (-2.1 - 1.33) | 6348 (3154-13132) | 5313 (3428-7768) | 61.53 (30.57-127.29) | 22.21 (14.33-32.48) | -3.38 (-3.64 - -3.12) |
| Ukraine | 107525 (85519-134461) | 55475 (43802-70430) | 715.94 (569.42-895.29) | 619.21 (488.91-786.12) | -0.35 (-0.58 - -0.12) | 8601 (5546-12903) | 3249 (1991-5227) | 57.27 (36.93-85.91) | 36.26 (22.22-58.34) | -1.44 (-1.85 - -1.04) |
| United Arab Emirates | 2170 (1685-2806) | 4346 (3352-5709) | 305.99 (237.51-395.62) | 286.74 (221.14-376.67) | -0.21 (-0.38 - -0.04) | 233 (159-324) | 357 (225-536) | 32.88 (22.43-45.69) | 23.56 (14.85-35.33) | -1.08 (-1.55 - -0.61) |
| United Kingdom | 101172 (78447-132301) | 80403 (62139-104663) | 679.35 (526.75-888.36) | 517.44 (399.9-673.57) | -0.91 (-0.98 - -0.84) | 7045 (3999-11570) | 5311 (3078-8658) | 47.31 (26.85-77.69) | 34.18 (19.81-55.72) | -1.08 (-1.17 - -0.99) |
| United Republic of Tanzania | 46546 (35296-61960) | 93483 (70199-126116) | 310.61 (235.54-413.47) | 304.52 (228.67-410.83) | -0.03 (-0.21 - 0.16) | 3629 (2364-5388) | 6907 (4420-10304) | 24.22 (15.78-35.96) | 22.5 (14.4-33.57) | -0.24 (-0.35 - -0.13) |
| United States of America | 287979 (226194-363235) | 229347 (173293-298257) | 390.4 (306.64-492.42) | 280.4 (211.87-364.65) | -1.29 (-2.08 - -0.48) | 15654 (8556-26106) | 12825 (7067-21663) | 21.22 (11.6-35.39) | 15.68 (8.64-26.49) | -1.17 (-1.74 - -0.59) |
| United States Virgin Islands | 259 (201-336) | 168 (126-222) | 623.76 (483.08-807.51) | 632.62 (474.39-839.35) | 0.15 (-0.14 - 0.43) | 18 (10-28) | 11 (6-17) | 42.37 (24.21-67.51) | 39.64 (21.41-66.03) | -0.22 (-0.46 - 0.01) |
| Uruguay | 7178 (5756-9044) | 6132 (4861-7712) | 666.76 (534.71-840.1) | 644.35 (510.77-810.28) | -0.11 (-0.15 - -0.07) | 704 (473-1004) | 438 (268-672) | 65.38 (43.96-93.29) | 46.04 (28.18-70.65) | -1.2 (-1.34 - -1.07) |
| Uzbekistan | 68365 (55323-85101) | 72873 (58190-92515) | 639.85 (517.79-796.49) | 554.26 (442.58-703.66) | -0.47 (-0.66 - -0.27) | 7214 (5066-10011) | 5880 (3942-8497) | 67.52 (47.41-93.7) | 44.72 (29.99-64.63) | -1.38 (-1.55 - -1.2) |
| Vanuatu | 161 (129-202) | 278 (221-352) | 195.97 (156.52-245.5) | 199.19 (158.5-252.41) | -0.06 (-0.89 - 0.77) | 37 (26-49) | 50 (35-67) | 44.85 (31.44-59.38) | 36.16 (25.22-48.05) | -0.6 (-1.18 - -0.02) |
| Venezuela (Bolivarian Republic of) | 71816 (57091-91895) | 67260 (53113-85212) | 789.85 (627.89-1010.67) | 723.29 (571.16-916.35) | -0.47 (-0.99 - 0.05) | 11781 (8286-15986) | 6452 (4157-9627) | 129.57 (91.13-175.82) | 69.38 (44.7-103.53) | -2.13 (-2.38 - -1.87) |
| Viet Nam | 92212 (73867-116634) | 79886 (63684-101850) | 276.5 (221.49-349.72) | 284.57 (226.85-362.81) | 0.06 (-0.27 - 0.39) | 15512 (10948-20707) | 7387 (4829-10699) | 46.51 (32.83-62.09) | 26.32 (17.2-38.11) | -1.91 (-2.06 - -1.75) |
| Yemen | 23904 (18446-30887) | 105787 (59199-208825) | 281.24 (217.02-363.4) | 648.42 (362.86-1279.99) | 2.97 (1.56 - 4.4) | 4750 (3066-7251) | 9100 (5154-16119) | 55.88 (36.07-85.31) | 55.78 (31.59-98.8) | 0.01 (-0.6 - 0.63) |
| Zambia | 14216 (10966-18663) | 26703 (19615-36108) | 301.98 (232.94-396.44) | 272.82 (200.4-368.91) | -0.29 (-0.42 - -0.17) | 1186 (788-1716) | 1964 (1227-2893) | 25.19 (16.74-36.46) | 20.07 (12.54-29.56) | -0.74 (-0.89 - -0.6) |
| Zimbabwe | 11011 (8722-13926) | 16452 (13288-20417) | 182.76 (144.77-231.14) | 215.88 (174.36-267.9) | 0.58 (0.18 - 0.99) | 987 (642-1437) | 1378 (906-1939) | 16.38 (10.65-23.85) | 18.08 (11.89-25.45) | 0.36 (0.23 - 0.49) |
